# Altered hippocampal gene expression, glial cell population, and neuronal excitability in aminopeptidase P1 deficiency

**DOI:** 10.1038/s41598-020-79656-6

**Published:** 2021-01-13

**Authors:** Sang Ho Yoon, Young-Soo Bae, Sung Pyo Oh, Woo Seok Song, Hanna Chang, Myoung-Hwan Kim

**Affiliations:** 1 Department of Physiology and Biomedical Sciences, Seoul National University College of Medicine, Seoul, 03080 Korea; 2 Neuroscience Research Institute, Seoul National University Medical Research Center, Seoul, 03080 Korea; 3 Seoul National University Bundang Hospital, Seongnam, 13620 Gyeonggi Korea

**Keywords:** Neuroscience, Cellular neuroscience, Diseases, Neurological disorders

## Abstract

Inborn errors of metabolism are often associated with neurodevelopmental disorders and brain injury. A deficiency of aminopeptidase P1, a proline-specific endopeptidase encoded by the Xpnpep1 gene, causes neurological complications in both humans and mice. In addition, aminopeptidase P1-deficient mice exhibit hippocampal neurodegeneration and impaired hippocampus-dependent learning and memory. However, the molecular and cellular changes associated with hippocampal pathology in aminopeptidase P1 deficiency are unclear. We show here that a deficiency of aminopeptidase P1 modifies the glial population and neuronal excitability in the hippocampus. Microarray and real-time quantitative reverse transcription-polymerase chain reaction analyses identified 14 differentially expressed genes (Casp1, Ccnd1, Myoc, Opalin, Aldh1a2, Aspa, Spp1, Gstm6, Serpinb1a, Pdlim1, Dsp, Tnfaip6, Slc6a20a, Slc22a2) in the Xpnpep1^**−/−**^ hippocampus. In the hippocampus, aminopeptidase P1-expression signals were mainly detected in neurons. However, deficiency of aminopeptidase P1 resulted in fewer hippocampal astrocytes and increased density of microglia in the hippocampal CA3 area. In addition, Xpnpep1^**−/−**^ CA3b pyramidal neurons were more excitable than wild-type neurons. These results indicate that insufficient astrocytic neuroprotection and enhanced neuronal excitability may underlie neurodegeneration and hippocampal dysfunction in aminopeptidase P1 deficiency.

## Introduction

One of the hallmarks of living systems is metabolism, the sum of biochemical processes that occur within each cell of a living organism, and many human diseases are associated with abnormal metabolic states^1^. Metabolic disturbance or perturbation in brain cells often causes neuropsychiatric disorders and severe brain injury. One example is inborn errors of metabolism (IEMs), also known as inherited metabolic disorders (IMDs), which frequently accompany abnormal neurodevelopment and behaviors, intellectual disability, epilepsy, and neurodegeneration^2,3^. IEMs are a large class of genetic disorders resulting from a deficiency or defective activity of a single enzyme in the metabolic pathway. Despite the substantial prevalence of IEMs ranging from 1/784 to 1/2555 births collectively^4^, each disorder is individually rare. This rarity hinders understanding of each disease and development of effective medications to relieve the symptoms of the disease, especially the neurological symptoms. Animal models for rare inheritable diseases provide opportunities to understand pathological mechanisms, including molecular and cellular changes associated with the disease.

Aminopeptidase P1, encoded by the Xpnpep1 gene, is a proline-specific endopeptidase that cleaves the N-terminal amino acid residue of peptides with a penultimate proline residue^5^. Aminopeptidase P1 deficiency is a rare inherited metabolic disease that causes massive urinary excretion of undigested peptides containing a penultimate proline^6,7^. In addition to peptiduria, Xpnpep1-mutant mice and a human patient with aminopeptidase P1 deficiency exhibit neurological complications such as microcephaly and epilepsy^6–8^. Aminopeptidase P1 is abundant in the brain and the expression of Xpnpep1 mRNA in the human brain tissue is as stable as housekeeping genes^9^, indicating that aminopeptidase P1 is critical for normal brain function. Consistent with this idea, deficiency of aminopeptidase P1 in mice results in reduced body and brain size, hyperactivity, impaired hippocampus-dependent learning, and neurodegeneration in the hippocampal CA3 area^7,8^. Deducing from the enzymatic activity of aminopeptidase P1, bioactive peptides with a penultimate proline such as bradykinin, substance P, neuropeptide Y, peptide YY, and interleukins, are thought to be the substrates of aminopeptidase P1^10^. Accumulation or delayed removal of peptides containing a penultimate proline may modify molecular signaling in the brain cells, resulting in brain dysfunction in aminopeptidase P1 deficiency. However, how deficiency of aminopeptidase P1 causes neurodegeneration and hippocampal dysfunction is unclear.

The present study focused on molecular and cellular alterations in the hippocampus with aminopeptidase P1 deficiency because proper metabolism of brain cells, including neurons, astrocytes, and microglia, is critical for normal brain function and because glial alteration may contribute to complex neurological and psychiatric complications in IEMs. We found that aminopeptidase P1 is predominantly expressed in neurons, and genetic ablation of Xpnpep1 resulted in enhanced neuronal excitability in CA3b pyramidal neurons, which may account for neurodegeneration and epilepsy in Xpnpep1^**−/−**^ mice. In addition, deficiency of aminopeptidase P1 modified gene expression as well as the population of glial cells in the hippocampus. We also show that glial changes in aminopeptidase P1 deficiency are distinct from changes seen in common late-onset neurodegenerative diseases and other IEMs.

## Results

### Gene expression profile of the hippocampus in mice with aminopeptidase P1 deficiency

Xpnpep1^**−/−**^ mice exhibit hippocampal pathology and impaired hippocampus-dependent learning and memory^8^. To identify differentially expressed genes in the Xpnpep1^**−/−**^ hippocampus, we performed microarray analysis of gene expression profiles in the hippocampal samples from 5-week-old wild-type (WT; Xpnpep1^+/+^) and Xpnpep1^**−/−**^ mice. To avoid possible false-positive interpretations caused by a variation in the individual sample contribution to the pooled sample, each sample was independently hybridized to a single microarray chip and the expression levels of each gene were compared among animals by statistical analyses^11^.

**Figure 1:**
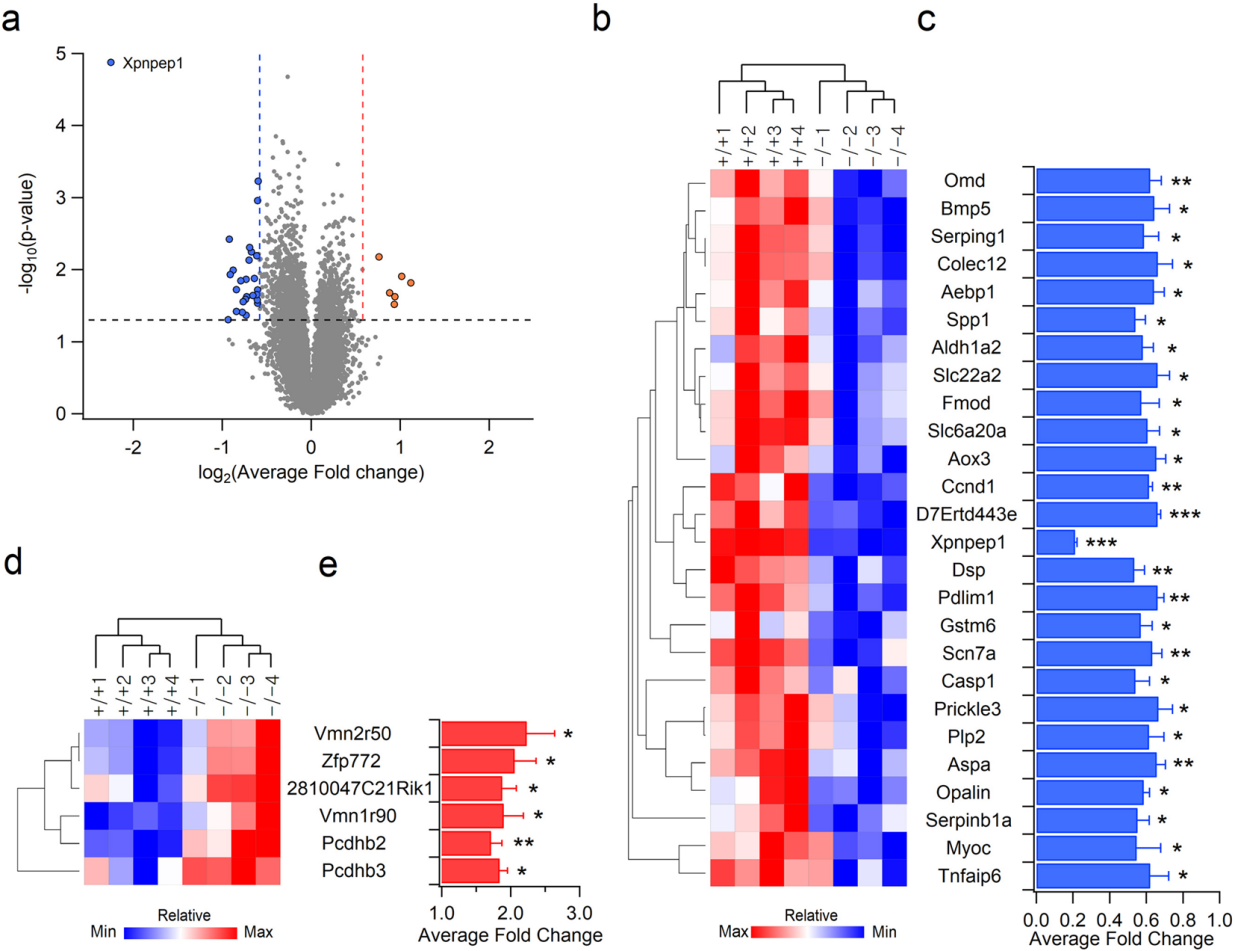
Microarray analysis of gene expression profiling in the Xpnpep1^−/−^ hippocampus. (**a**) Volcano plot showing the fold change and *p* value of the individual probe sets by microarray analysis. The vertical lines indicate 1.5-fold up-regulation (dotted red line) and down-regulation (dotted blue line), respectively. The horizontal dotted line represents the cutoff significance level (*p* = 0.05). The red and blue dots indicate up-regulated and down-regulated genes, respectively, with statistical significance. (**b**) The down-regulated genes in the Xpnpep1^−/−^ hippocampus are visualized by heatmap and hierarchical clustering. Each gene and sample were clustered by similarity between the expression patterns of genes. Red indicates high relative expression and blue indicates low relative expression. (**c**) Bar graphs represent average fold changes of down-regulated genes in the Xpnpep1^−/−^ hippocampus. (**d**,**e**) Treeview and hierarchical clustering (**d**) and average fold changes (**e**) of up-regulated genes in the Xpnpep1^−/−^ hippocampus. (**b**,**d**) Male: +/+1, +/+4, −/− 1, and −/−4; female: +/+2, +/+3, −/−2, and −/−3. (**c**,**e**) *p < 0.05; **p < 0.01; ***p < 0.001 by Student’s *t*-test, n = 4 pairs.

Of the total 28,853 gene-level probe sets, the number of probe sets considered to be valid for individual samples did not differ between genotypes (+/+, 27,856 ± 84; −/−, 28,093 ± 90; t_(6)_ =  − 1.926, *p* = 0.102). However, when we compared the expression levels of each gene with a threshold fold change greater than 1.5, or less than 0.667 (1.5-fold down-regulation), we identified 32 genes as differentially expressed genes (DEGs) with statistical significance (*p* < 0.05, Fig. 1a). Among the 32 DEGs (Table 1), 26 genes (Omd, Bmp5, Serping1, Colec12, Aebp1, Spp1, Aldh1a2, Slc22a2, Fmod, Slc6a20a, Aox3, Ccnd1, D7Ertd443e, Xpnpep1, Dsp, Pdlim1, Gstm6, Scn7a, Casp1, Prickle3, Plp2, Aspa, Opalin, Serpinb1a, Myoc, and Tnfaip6) were down-regulated (Fig. 1b,c), while 6 genes (Vmn2r50, Zfp772, 2810047C21Rik1, Vmn1r90, Pcdhb2, and Pcdhb3) were up-regulated in the Xpnpep1^**−/−**^ hippocampus (Fig. 1d,e). Notably, Xpnpep1 exhibited the largest fold changes (less than 0.21, *p* = 0.0000133) in gene expression by microarray. Since the Xpnpep1 gene in the mutant mice was disrupted by gene trap mutagenesis to produce Xpnpep1-β-geo (β-galactosidase + neomycin phosphotransferase) fusion transcripts^7^ which lack the coding region of the peptidase domain, weak Xpnpep1-positive signals in the Xpnpep1^**−/−**^ samples might stem from probes in the Xpnpep1 probe set that detect upstream endogenous Xpnpep1 regions of the Xpnpep1-β-geo fusion transcript. Interestingly, the expression levels of Slc6a20a (solute carrier family 6, member 20; sodium/imino-acid transporter, SIT1) were significantly lower in Xpnpep1^**−/−**^ than in the WT samples (Fig. 1c). Considering that Slc6a20a transports imino acids, such as proline and hydroxyproline, across the plasma membrane through the Na^+^-dependent mechanism^12,13^, downregulation of Slc6a20a may reflect homeostatic responses of hippocampal cells to altered imino acid metabolism in Xpnpep1^**−/−**^ mice. In addition, reduced expression of Dsp indicates that postnatal development of the dentate gyrus is delayed in Xpnpep1^**−/−**^ mice^14^.

**Table 1:** List of DEGs in Xpnpep1^−/−^ hippocampus identified by microarray analysis.

Gene symbol	Full gene name
Vmn2r50	Vomeronasal 2, receptor 50A
Zfp772	Zinc finger protein 772
2810047C21Rik1	RIKEN cDNA 2810047C21 gene 1
Vmn1r90	Vomeronasal 1 receptor 90
Pcdhb2	Protocadherin beta 2
Pcdhb3	Protocadherin beta 3
Omd	Osteomodulin
Bmp5	Bone morphogenetic protein 5
Serping1	Serine (or cysteine) peptidase inhibitor, clade G, member 1
Colec12	Collectin sub-family member 12
Aldh1a2	Aldehyde dehydrogenase family 1, subfamily A2
Slc22a2	Solute carrier family 22 (organic cation transporter), member 2
Fmod	Fibromodulin
Slc6a20a	Solute carrier family 6 (neurotransmitter transporter), member 20A
Aox3	Aldehyde oxidase 3
Aebp1	AE binding protein 1
Spp1	Secreted phosphoprotein 1
Gstm6	Glutathione S-transferase, mu 6
Scn7a	Sodium channel, voltage-gated, type VII, alpha
Ccnd1	Cyclin D1
D7Ertd443e	DNA segment, Chr 7, ERATO Doi 443, expressed
Xpnpep1	X-prolyl aminopeptidase (aminopeptidase P) 1, soluble
Dsp	Desmoplakin
Pdlim1	PDZ and LIM domain 1 (elfin)
Casp1	Caspase 1
Prickle3	Prickle planar cell polarity protein 3
Plp2	Proteolipid protein 2
Serpinb1a	Serine (or cysteine) peptidase inhibitor, clade B, member 1a
Aspa	Aspartoacylase
Opalin	Oligodendrocytic myelin paranodal and inner loop protein
Myoc	Myocilin
Tnfaip6	Tumor necrosis factor alpha induced protein 6

### Real-time quantitative reverse transcription PCR (qRT-PCR) confirmed altered gene expression in hippocampal neurons and glial cells

To validate the alterations in gene expression observed in the microarray, we further examined the mRNA levels of the 31 DEGs, except Xpnpep1, by real-time qRT-PCR, using a different cohort of mice. Primers for each gene were designed to generate 120–250 bp products at pre-determined annealing temperatures (Supplementary Table 1), and hippocampal cDNA was prepared from 5 independent WT and Xpnpep1^**−/−**^ mice (4–5 weeks of age). Expression levels of each target gene were determined by the ratio to the reference gene, Gapdh.

**Figure 2:**
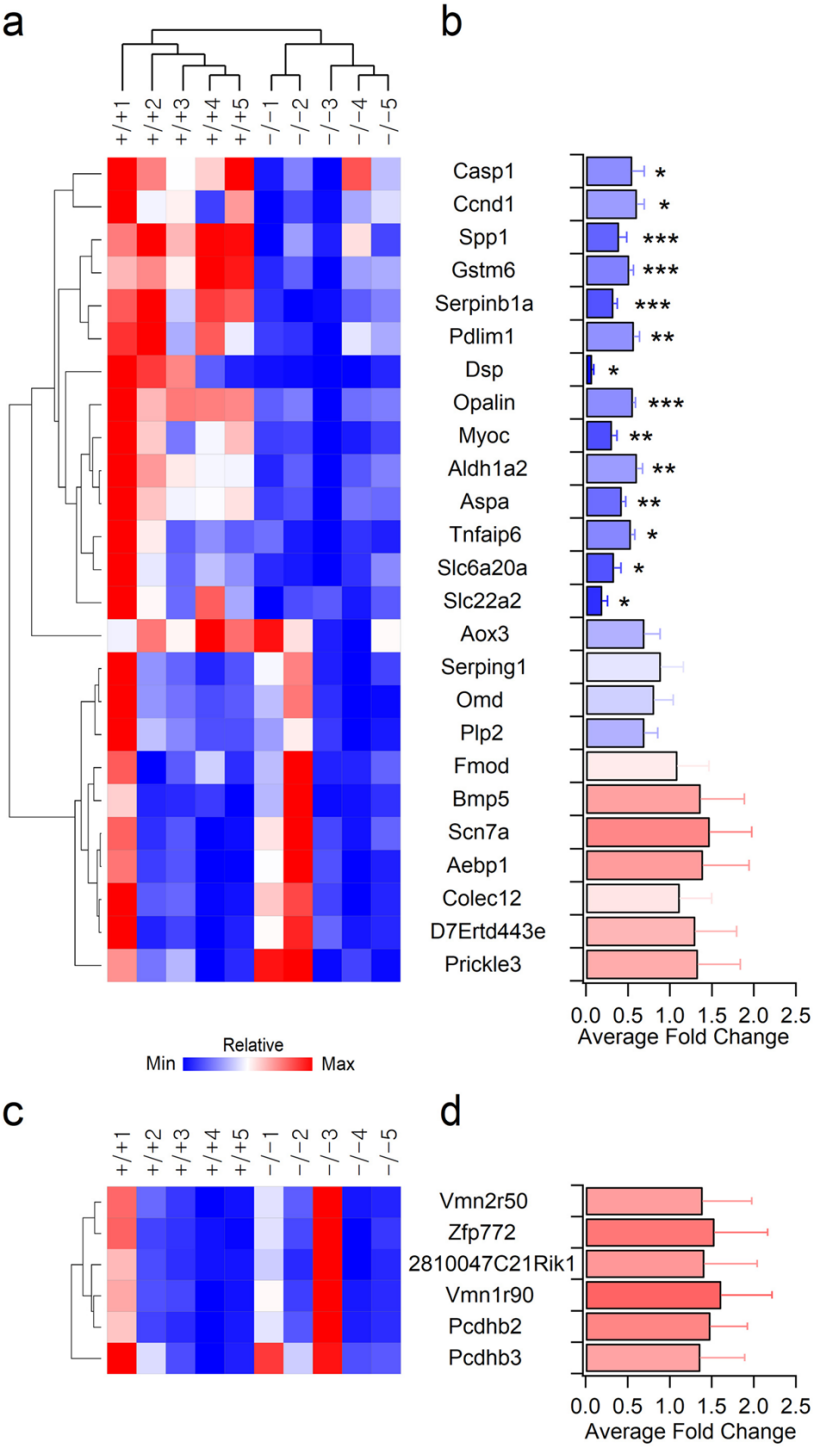
Validation of microarray results by quantitative reverse transcription PCR (qRT-PCR) (**a**,**b**) qRT-PCR validation of 25 down-regulated genes identified by microarray analysis. (**a**) Genes with similar expression patterns were clustered and visualized by Treeview. (**b**) Average fold changes in expression levels of genes identified as down-regulated genes from microarray analysis were analyzed by qRT-PCR. *p < 0.05; **p < 0.01; ***p < 0.001 by Student’s *t*-test, n = 5 pairs. (**c**,**d**) mRNA expression levels of genes identified as up-regulated genes from microarray analysis were not statistically different between genotypes. Treeview representation (**c**) and average fold changes (**d**) of expression determined by qRT-PCR.

When we examined the expression levels of 25 down-regulated genes identified from microarray analysis by qRT-PCR, mRNA levels of 14 genes (Casp1, Ccnd1, Spp1, Gstm6, Serpinb1a, Pdlim1, Dsp, Opalin, Myoc, Aldh1a2, Aspa, Tnfaip6, Slc6a20a, and Slc22a2) in the hippocampus were significantly reduced in the Xpnpep1^**−/−**^ mice (male: −/− 2, −/− 3, −/− 5; female: −/− 1, −/− 4) compared to WT (male: +/+ 1, +/+ 4; female: +/+ 2, +/+ 3, +/+ 5) mice, while hippocampal expression levels of 11 genes (Aox3, Serping1, Omd, Plp2, Fmod, Bmp5, Scn7a, Aebp1, Colec12, D7Ertd443e, and Prickle3) were not different between genotypes (Fig. 2a,b). Similarly, qRT-PCR analysis of the up-regulated genes showed a different pattern when compared with the microarray analysis. Xpnpep1^**−/−**^ mice showed a tendency to increase the mRNA levels of the 6 genes (Vmn2r50, Zfp772, 2810047C21Rik1, Vmn1r90, Pcdhb2, and Pcdhb3), but the difference was not statistically significant (Fig. 2c,d). Since microarray and qRT-PCR data were obtained from different sets of animals, the 14 down-regulated genes confirmed by both microarray and qRT-PCR analyses were reliable DEGs associated with pathologic changes in the Xpnpep1^**−/−**^ hippocampus. Importantly, these DEGs comprise genes that are abundant in astrocytes (Myoc, Aldh1a2, Gstm6, Ccnd1, Tnfaip6)^15–19^, oligodendrocytes (Serpinb1a, Aspa, Opalin)^18^, microglia (Slc6a20a, Spp1)^12,20,21^ or neurons (Dsp, Slc22a2)^22–24^, indicating that a deficiency of aminopeptidase P1 affects both glia and neurons in the hippocampus.

### Expression patterns of aminopeptidase P1 in the hippocampus

Aminopeptidase P1 is widely expressed in brain neurons, including hippocampal principal neurons^7^. However, glial expression and subcellular distribution of aminopeptidase P1 in the hippocampus are unclear. Because our anti-aminopeptidase P1 antibody was not suitable for immunohistochemical staining and Xpnpep1 mutant mice express a β-galactosidase (lacZ) reporter under the control of the Xpnpep1 promoter, we labeled aminopeptidase P1-expressing cells in the hippocampal sections from 4 to 5-week-old Xpnpep1^+/−^ and Xpnpep1^−/−^ mice by X-gal staining. The sections were then immunostained with the neuronal marker NeuN, the astrocyte markers GFAP (glial fibrillary acidic protein) and S100β (S100 calcium-binding protein beta chain), the microglial marker Iba1 (ionized calcium binding adapter molecule 1), or the oligodendrocyte marker O4. In confocal fluorescence microscopy, we observed that each marker labeled distinct cell populations (Supplementary Fig. 1).

**Figure 3:**
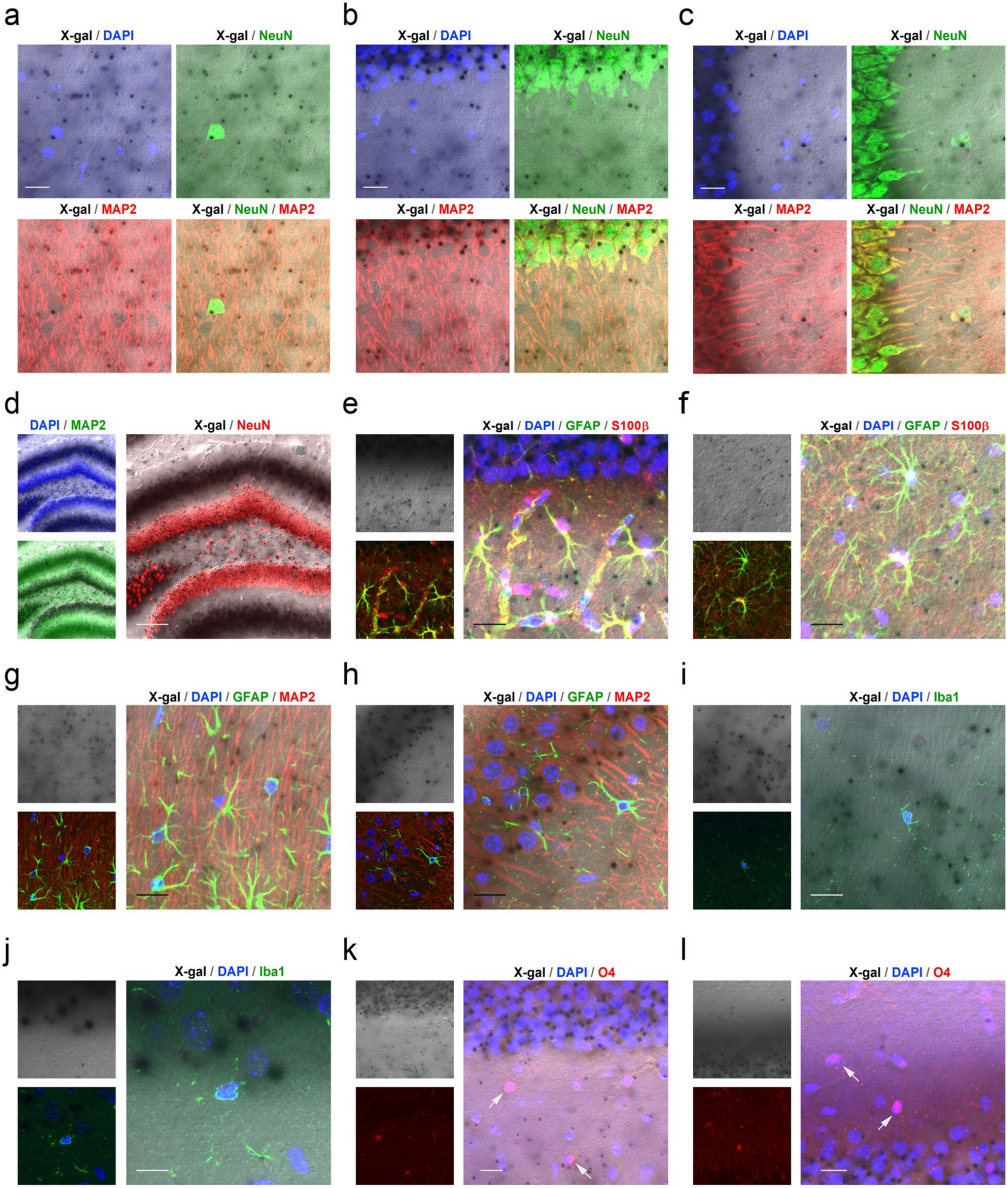
X-gal staining and immunohistochemistry revealed predominant neuronal expression of aminopeptidase P1 in the Xpnpep1^+/−^ hippocampus. (**a**) Transmitted image of X-gal inclusions in the Xpnpep1^+/−^ CA1 *stratum radiatum* region was merged with the fluorescence image of DAPI, NeuN, and/or MAP2. (**b**,**c**) Combined transmission and fluorescence of images show the presence of X-gal precipitates in the somata and dendrites of Xpnpep1^+/−^ CA1 (**b**) and CA3 (**c**) neurons. (**d**) X-gal precipitates (black) were detected in the GC layer, hilus (polymorphic layer), and outer molecular layer of DG (left). Right, X-gal signals overlapped well with NeuN immunoreactive signals in the DG hilus. (**e**,**f**) Left, astrocytes in CA1 (**e**), and CA3 (**f**) *stratum radiatum* from the X-gal (top) stained sections were identified by immunohistochemical staining with GFAP and S100β (bottom). X-gal signals are rarely found in the cell body and processes of Xpnpep1^+/−^ astrocytes (right). (**g**,**h**) Cell nuclei, neuronal dendrites, and astrocytic processes in the CA1 (**g**) and CA3 (**h**) areas from X-gal stained sections were labeled with DAPI, anti-MAP2, and anti-GFAP antibodies, respectively (left). Right, punctate X-gal signals in the CA1 *stratum radiatum* (**g**) and CA3 *stratum lucidum* (**h**) were predominantly localized in the MAP2-positive dendrites, but they were occasionally detected in GFAP-positive astrocyte processes. (**i**,**j**) Sections were stained with X-gal (left, top), and microglia located in the CA1 (**i**) and CA3 (**j**) *stratum radiatum* were immunostained with Iba1 (left, bottom). The merged images (right) show the absence of β-galactosidase activity in Xpnpep1^+/−^ microglia. (**k**,**l**) After X-gal staining (left, top), oligodendrocytes in the CA1 *stratum radiatum* (**k**) and *stratum oriens* (**l**) were immunolabeled with anti-O4 antibodies (left, bottom). Right, X-gal signals were not observed in the O4-positive cells (arrows). (**a**–**l**) DAPI was used to label the nuclei of neurons and glia. Scale bars: 10 µm (**j**), 20 µm (**a**–**c**,**e**–**i**,**k**,**l**), and 100 µm (**d**).

Consistent with previous results^7^, the hippocampal principal cell layers, which contain mainly excitatory neurons, exhibited intense X-gal staining signals by light transmission microscopy (Supplementary Fig. 2a–d). Strong punctate signals were mostly detected within the cell body of principal neurons in both Xpnpep1^+/−^ and Xpnpep1^−/−^ mice. In addition, we detected disperse X-gal-positive signals in the dentate gyrus (DG) hilus and outer molecular layer, CA3 *stratum lucidum,* and CA1 *stratum radiatum*. (Supplementary Fig. 2b–d). When we immunostained the X-gal stained section with NeuN antibodies, we observed X-gal precipitates within the NeuN-positive neuronal somata (Fig. 3a–d and Supplementary Fig. 2g–i). The merged transmittance and fluorescence images further revealed that X-gal signals were present in MAP2-positive dendrites of neurons in the hippocampus (Fig. 3a–d). However, X-gal signals scarcely overlapped with the astrocyte markers GFAP and S100β (Fig. 3e–h). In addition, X-gal signals were rarely detected in the CA3 *stratum radiatum*, CA1 *stratum lacunosum-moleculare*, and *stratum oriens* of CA1 and CA3, whereas astrocytes were abundant in these areas (Supplementary Fig. 3). Similar to astrocytes, X-gal signals did not overlap with microglia (Fig. 3i,j) and oligodendrocytes (Fig. 3k,l) markers.

Previous RNA sequencing studies reported that Xpnpep1 transcripts were present in astrocytes, microglia, and neurons^18,25^. However, when we examined the protein expression levels of aminopeptidase P1 in hippocampal neuron cultures and glial cultures prepared from Xpnpep1^+/+^ embryonic mice, the amount of aminopeptidase P1 was significantly higher (> 7 times) in the βIII-tubulin enriched neuronal lysates than in the GFAP-enriched glial lysates (Supplementary Fig. 4). Collectively, these results suggest that aminopeptidase P1 is predominantly expressed in neurons in the hippocampus.

**Figure 4:**
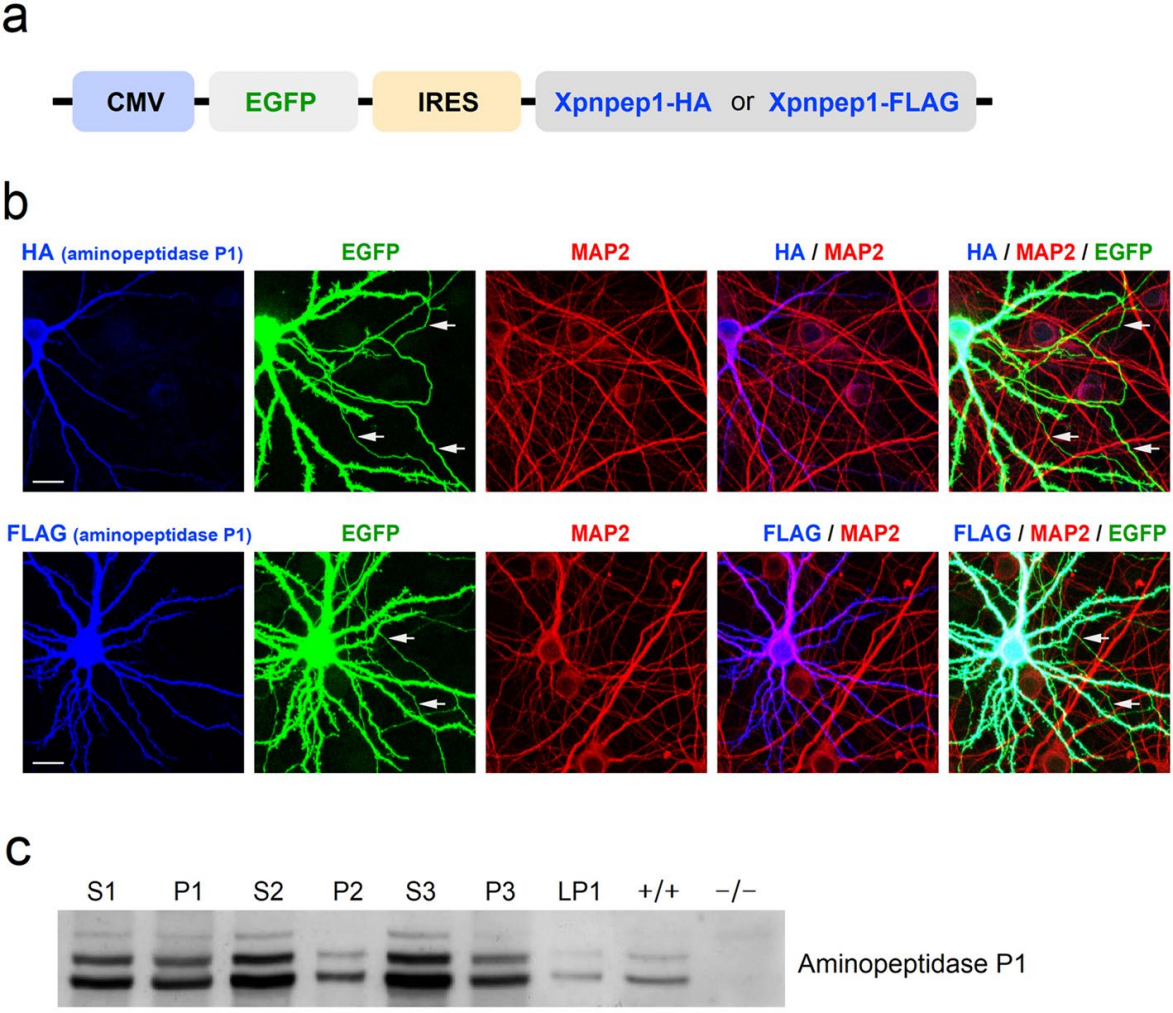
Cellular localization of aminopeptidase P1 in hippocampal neurons. (**a**) Schematic diagram of the bicistronic expression vector. (**b**) Cultured hippocampal neurons were transfected with the bicistronic expression vector using a calcium phosphate method. The dendrites of transfected (EGFP-positive) and neighboring untransfected (EGFP-negative) neurons were visualized by MAP2. Exogenously expressed aminopeptidase P1 protein was mainly distributed in the soma and dendrites of cultured hippocampal neurons. Arrows indicate MAP2-negative axons of transfected neurons. Scale bars, 20 μm. (**c**) Distribution of aminopeptidase P1 in subcellular brain fractions. Endogenous aminopeptidase P1 was detected in all fractions, with the strongest signal observed in the cytosolic fractions (S2 and S3). Whole brain homogenates from WT and Xpnpep1^–/–^ mice were used to determine the specificity of the aminopeptidase P1 antibody. S1, supernatant after P1 sedimentation; P1, crude nuclear fraction; S2, supernatant after P2 sedimentation; P2, crude synaptosomal pellet; S3, cytosolic; P3, light membranes; LP1, synaptosomal membranes.

X-gal signals in the hippocampal sections from Xpnpep1-mutant mice are produced by Xpnpep1-β-geo fusion proteins rather than endogenous aminopeptidase P1, indicating that the signal represents aminopeptidase P1-expressing cells but not the subcellular localization of aminopeptidase P1. As X-gal signals were mostly detected in the soma and dendrites of neurons, we further investigated the subcellular localization of aminopeptidase P1 in neurons by co-expressing FLAG- or HA-tagged aminopeptidase P1 with EGFP in cultured hippocampal neurons. To avoid nonspecific localization of aminopeptidase P1 caused by overexpression, we used the IRES (internal ribosome entry site)-containing bicistronic vector (Fig. 4a) because IRES-dependent gene expression is significantly weaker than cap-dependent expression^26^. In contrast to EGFP, which was distributed extensively throughout the transfected neurons, aminopeptidase P1 expressed by IRES-mediated translation was mainly detected in cell bodies and dendrites, but not in MAP2-negative axonal regions in the transfected neurons (Fig. 4b). Consistent with this result, endogenous aminopeptidase P1 was more distributed in the cytosolic fractions of hippocampal lysates (Fig. 4c). These results indicate that aminopeptidase P1 is a cytosolic protein that is mainly distributed in the somato-dendritic compartments of neurons.

### Deficiency of aminopeptidase P1 modifies the population of glial cells in the hippocampus

**Figure 5:**
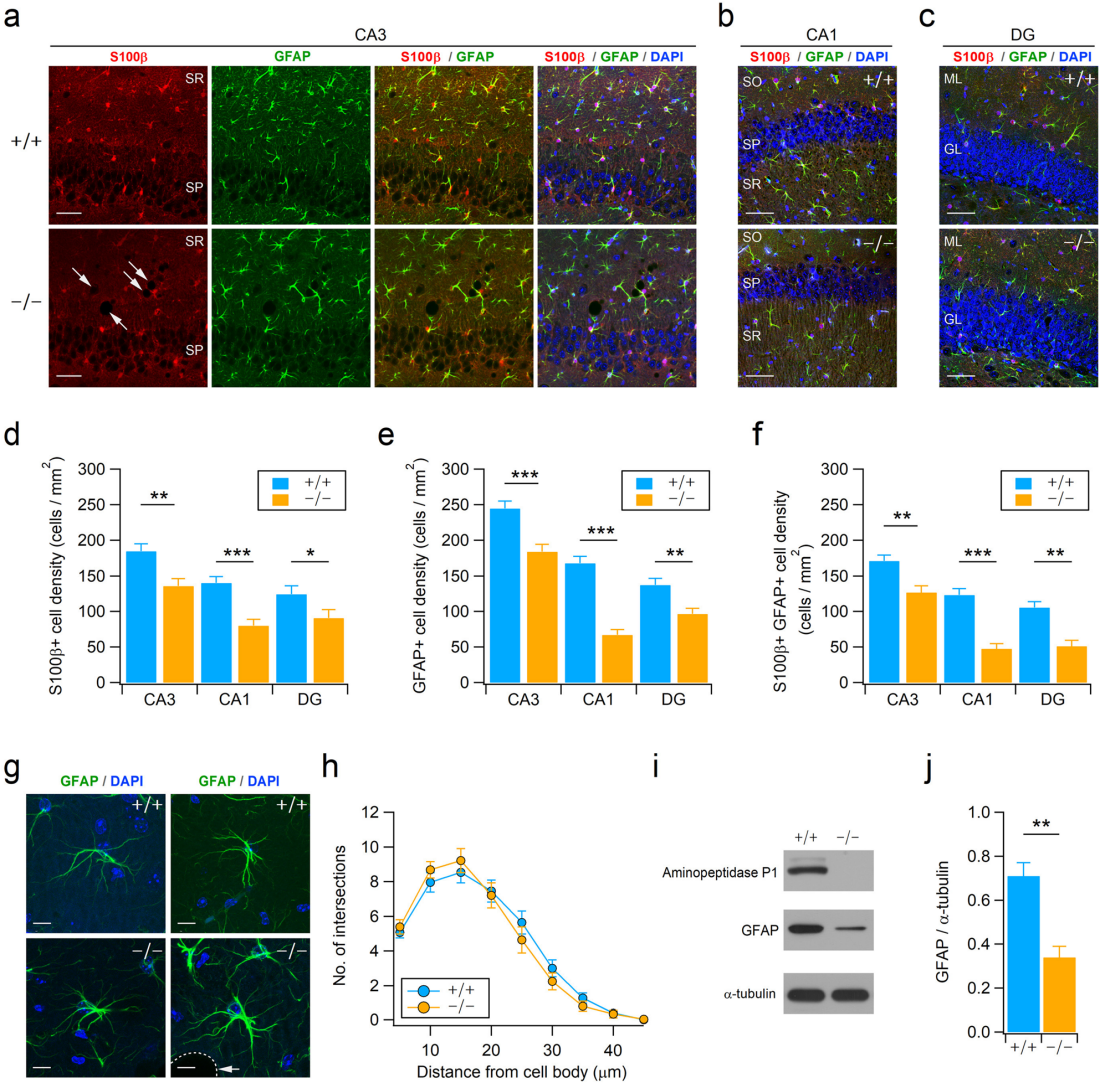
Reduction of astrocytes in the Xpnpep1^−/−^ hippocampus. (**a**) Astrocytes in the hippocampal CA3 regions from 4- to 5-week-old WT (top) and Xpnpep1^**−/−**^ (bottom) mice were stained with GFAP and S100β antibodies. DAPI was used to visualize cell nuclei, and arrows indicate vacuoles. Immunofluorescence images of hippocampal CA3 regions show fewer astrocytes in Xpnpep1^**−/−**^ mice than in WT mice. (**b**,**c**) Astrocytes in the CA1 (**b**) and DG (**c**) subfields of Xpnpep1^+/+^ (top) and Xpnpep1^**−/−**^ (bottom) mice are visualized. Scale bars, 50 µm (**a**–**c**). SP, *stratum pyramidale*; SR, *stratum radiatum*; SO, *stratum oriens*; ML, molecular layer; GL, granule cell layer. (**d**–**f**) The density of GFAP- and S100β-positive cells in the CA3, CA1, and DG regions were lower in Xpnpep1^**−/−**^ mice than in WT mice. *p < 0.05; **p < 0.01; ***p < 0.001 by Student’s *t*-test, n = 6 slices from 3 mice for each genotype. (**g**) The morphology of astrocytes and cell nuclei in the hippocampal CA3 subfield were visualized by GFAP (green) and DAPI (blue), respectively. The morphological features of reactive astrocytes, such as hypertrophy and extension of processes, were not detected in Xpnpep1^**−/−**^ astrocytes. Arrow indicates vacuole. Scale bars, 10 µm. (**h**) Sholl analysis of astrocyte complexity in the hippocampal CA3 subfield. n = 28 cells from 3 mice per genotype. *p* > 0.05 by Student’s *t*-test and Mann–Whitney test. (**i**) Representative western blots showing the expression levels of aminopeptidase P1 and GFAP in the hippocampal extracts. Western blotting using anti-α-tubulin antibody was performed to ensure equal protein loading and transfer, and quantification of protein levels in each sample. (**j**) Quantification of GFAP protein levels (right) in the Xpnpep1^+/+^ (n = 4) and Xpnpep1^**−/−**^ (n = 4) hippocampus. t_(6)_ = 4.62, **p = 0.0036 by Student’s *t*-test.

Despite exhibiting predominant expression of aminopeptidase P1 in neurons, DEGs identified by microarray and qRT-PCR indicate that aminopeptidase P1 deficiency affects glial cells in the hippocampus. Since astrocyte alterations are frequently associated with brain disorders^27^, we first determined the density of astrocytes in the hippocampus of 5-week-old mice using the astrocyte markers GFAP and S100β. GFAP expression in astrocytes is known not to be uniform. It is strong in astrocytes compartmentalized to the hippocampus or reactive astrocytes, while astrocytes in other brain areas exhibit mild to weak GFAP expression in the normal state^28,29^. S100β is less specific than GFAP, but it is broadly expressed in astrocytes^30^. Despite that both genotypes showed similar cross-sectional areas in the hippocampus (Supplementary Fig. 5a and b), Xpnpep1^**−/−**^ mice exhibited fewer GFAP-positive cells in the hippocampus compared to WT mice (Fig. 5a–f). Similarly, the number of S100β-positive cells was decreased in the Xpnpep1^**−/−**^ hippocampus. Quantification of the numbers of GFAP- or S100β-positive cells revealed significant differences in the density of hippocampal astrocytes between the two genotypes (Fig. 5d–f). Notably, a reduction in astrocyte density was detected in the whole hippocampal subregions including CA3, CA1, and DG areas (Fig. 5a–f). Considering that many neurodegenerative diseases accompany reactive astrogliosis and that reduced astrocyte density was observed in major depressive disorder and starvation^27,31^, neurodegeneration accompanying astrocyte reduction in the hippocampus is a unique pathological change in aminopeptidase P1 deficiency. In addition, the morphology of astrocytes in the Xpnpep1^**−/−**^ CA3 subfields, determined by GFAP-immunoreactive signals distributed in the cellular processes, was similar to that of control astrocytes (Fig. 5g), despite the presence of vacuoles of varying sizes in the Xpnpep1^**−/−**^ CA3 subfields (Fig. 5a,g). Sholl analyses of astrocytic processes showed no difference between the two genotypes (Fig. 5h). This observation also eliminates the possibility of astrocytic gliosis in the Xpnpep1^**−/−**^ hippocampus. Consistent with the reduced density of astrocytes, protein expression levels of GFAP were significantly decreased in the Xpnpep1^**−/−**^ hippocampus (Fig. 5i,j). However, there is a possibility that GFAP expression in each astrocyte, in addition to the reduced astrocyte density, was reduced by the deficiency of aminopeptidase P1.

**Figure 6:**
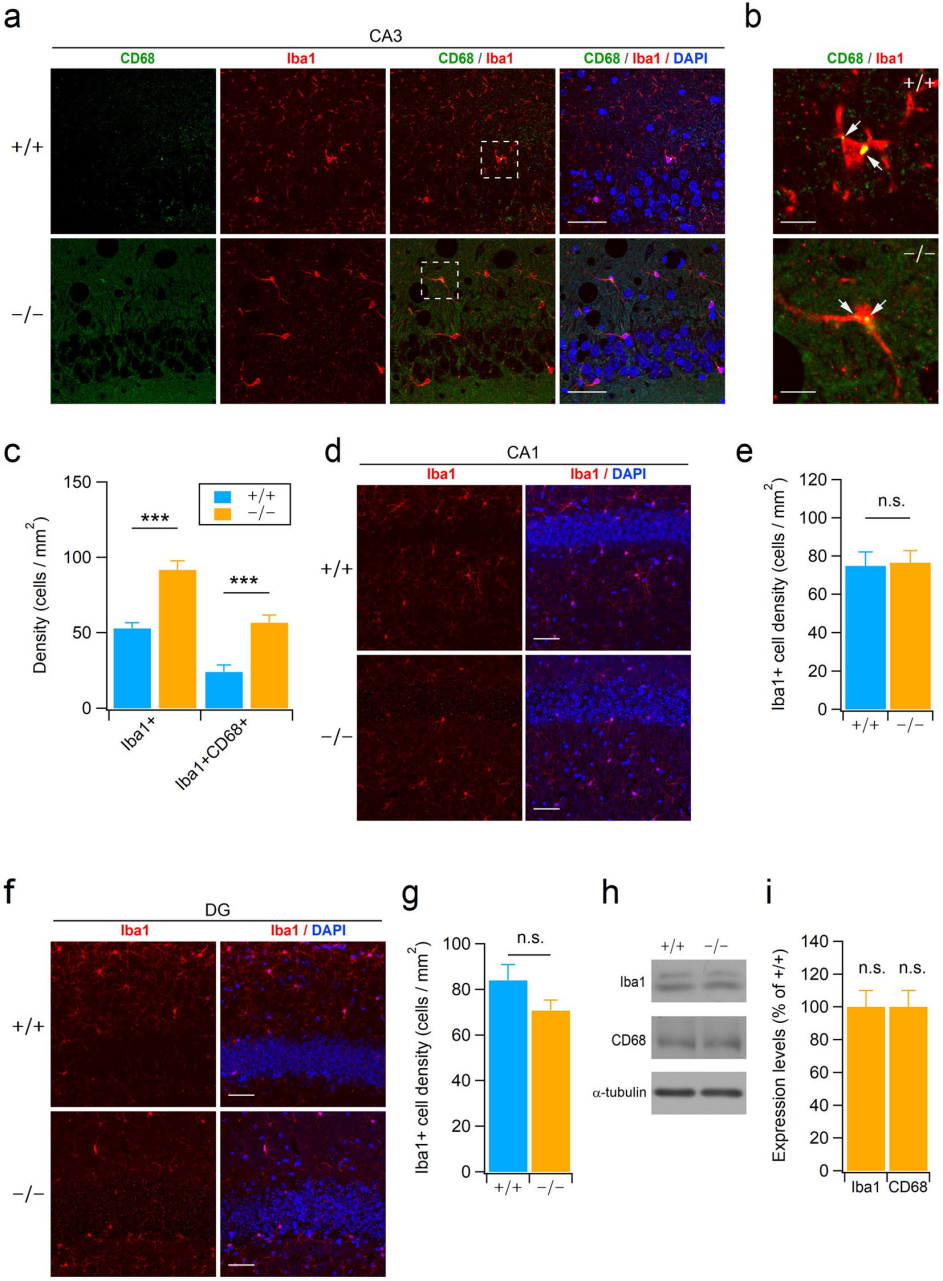
The density of microglia was increased in the Xpnpep1^−/−^ CA3 area. (**a**) Representative images of hippocampal CA3 regions stained with CD68 and Iba1 antibodies showing a higher number of microglia in Xpnpep1^**−/−**^ (bottom) than WT (top) mice. Scale bars, 50 µm. (**b**) Higher magnification views of areas indicated by the dotted white box in panel (**a**) show morphological features of normal resting microglia in both Xpnpep1^**+/+**^ (top) and Xpnpep1^**−/−**^ (bottom) mice. Scale bars, 10 µm. Note the punctate immunoreactive signals of CD68 (arrows) near the cell body but not the processes of microglia. (**c**) Increased density of microglia in the Xpnpep1^**−/−**^ CA3 region. n = 6 slices from 3 mice per genotype. U = 82 (Iba1+) and 102.5 (Iba1+CD68+), Z =  − 4.39 (Iba1+) and − 3.97 (Iba1+CD68+), ***p < 0.001 by Mann–Whitney test. (**d**) Microglia in the hippocampal CA1 area were immunostained with Iba1 antibodies (left) and merged (right) with DAPI signals. Scale bars, 50 µm. (**e**) Quantification of Iba1-positive cell density in hippocampal CA1 subfields. n.s., not significant. t_(10)_ =  − 0.17, *p* = 0.87 by Student’s *t*-test. (**f**) Iba1-immnostained (left) and merged (right) images with DAPI staining showing normal density of microglia in the Xpnpep1^**−/−**^ (bottom) DG subfield. Scale bars, 50 µm. (**g**) The density of microglia in the hippocampal DG is not changed by aminopeptidase P1 deficiency. n.s., not significant. t_(10)_ = 1.59, *p* = 0.13 by Student’s *t*-test. (**h**,**i**) Western blot images (**h**) and quantification (**i**) of Iba1 and CD68 proteins in the hippocampal lysates. n = 4 pairs. α-tubulin was used as a loading control for western blotting. t_(6)_ =  − 0.21 (Iba1) and − 0.81 (CD68), *p* = 0.84 (Iba1) and 0.45 (CD68) by Student’s *t*-test. n.s., not significant.

We next examined microglial populations in the hippocampus using Iba1 and CD68 antibodies. Because Xpnpep1^**−/−**^ mice exhibit microcephaly^7^, we selected coronal brain sections that displayed similar hippocampus morphology and size for both genotypes (Supplementary Fig. 5c and d). Iba1-positive signals were detected in the cell body and thin processes of microglia, whereas immunoreactive signals for CD68, the reactive microglia marker^32^, were mainly detected as tiny puncta near the cell bodies but not entire cell structures in both genotypes of mice (Fig. 6a,b). When we induced microglial activation in WT mice by intraperitoneal injections of lipopolysaccharide (LPS, 1 µg/kg; once daily for 4 days), intense immunoreactivity for CD68 was detected in the cell body and processes of microglia (Supplementary Fig. 6). Punctate CD68-positive signals in Xpnpep1^**−/−**^ microglia suggest that deficiency of aminopeptidase P1 does not induce activation of microglia in the hippocampus (Fig. 6a,b). However, the density of microglia in the CA3 subfields of Xpnpep1^**−/−**^ mice was significantly higher than those in WT mice (Fig. 6a,c). Interestingly, both genotypes showed similar numbers of Iba1-positive cells in the DG and CA1 subfields of the hippocampus (Fig. 6d–g). This finding is consistent with the neuropathology of Xpnpep1^**−/−**^ mice in that neurodegeneration was exclusively observed in the CA3 subfield^8^. In addition, some microglia were present but did not accumulate around vacuoles in the CA3 area of Xpnpep1^**−/−**^ mice (Fig. 6a and Supplementary Fig. 7)^33^. When we examined the expression levels of Iba1 and CD68 proteins in the whole hippocampal extracts, the expression levels of these proteins were not significantly different between the two genotypes (Fig. 6h,i). Collectively, these results suggest that a deficiency of aminopeptidase P1 selectively increases the number of microglial cells in the hippocampal CA3 subfield.

**Figure 7:**
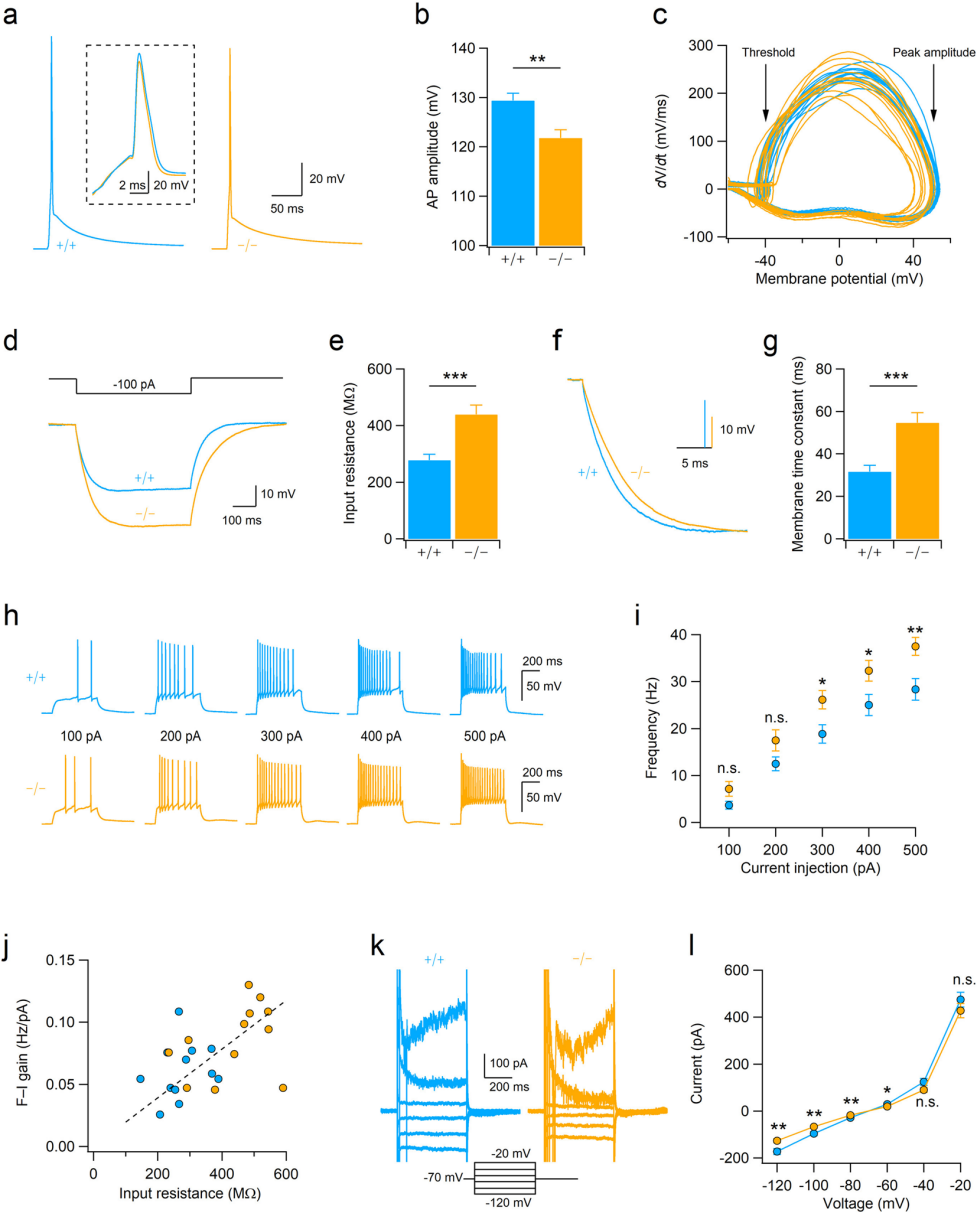
Hyperexcitability of CA3b pyramidal neurons in mice lacking aminopeptidase P1. (**a**) Mean traces of APs (n = 12 for each genotype) recorded in the CA3b pyramidal neurons. Inset, enlarged traces showing the peak amplitudes and time course of AP spikes. (**b**) Bar graphs represent the amplitude of APs in CA3b neurons. t_(22)_ = 3.386, **p = 0.003 by Student’s *t*-test. (**c**) First order derivatives (dV/dt) of the membrane potential during the action potential were calculated from WT (n = 12, blue traces) and Xpnpep1^**−/−**^ (n = 12; orange traces) CA3b neurons and plotted as a function of membrane voltage. (**d**) Sample traces of voltage response to hyperpolarizing current injection (500 ms) in CA3b pyramidal neurons. (**e**) Bar graphs represent the input resistance of CA3b pyramidal neurons measured by the hyperpolarizing step shown in (**d**). t_(22)_ =  − 4.15, ***p < 0.001 by Student’s *t*-test. (**f**) Voltage responses shown in (**d**) were normalized to their maximum amplitude, and the initial portion of the responses is shown with an expanded time scale. (**g**) Increased membrane time constant in Xpnpep1^**−/−**^ CA3b neurons. t_(22)_ =  − 4.10, ***p < 0.001 by Student’s *t*-test. (**h**) Sample traces of APs recorded in CA3 neurons in response to depolarizing current pulses (100–500 pA, 500 ms). (**i**) Averaged F–I curves in CA3 pyramidal neurons from WT (n = 12 from 3 animals) and Xpnpep1^**−/−**^ (n = 12 from 3 animals) mice. n.s., not significant (*p* > 0.05); *p < 0.05; **p < 0.01 by Student’s *t*-test. (**j**) The slopes of the F–I relationship measured between 100 and 300 pA of input current were plotted against R_in_ of each cell. The dashed line represents the best fit for the linear relation between the F–I gain and R_in_. (**k**) Sample traces of membrane currents measured from WT and Xpnpep1^**−/−**^ CA3b neurons. Inset, Voltage steps elicited from a holding potential of − 70 mV in 20 mV increments from − 120 to − 20 mV. (**l**) Current–voltage (I–V) relationship for net membrane current at the end of the voltage step recorded from Xpnpep1^**+/+**^ (n = 13 cells from 3 animals) and Xpnpep1^**−/−**^ (n = 13 cells from 3 animals) CA3b neurons. n.s., not significant (*p* > 0.05); *p < 0.05; **p < 0.01 by Student’s *t*-test.

### Aminopeptidase P1-deficiency enhances excitability of CA3 pyramidal neurons

Xpnpep1^**−/−**^ mice exhibit neurodegeneration and vacuolation in the CA3 area, but the pattern of alteration in the glial population observed in the Xpnpep1^**−/−**^ hippocampus is quite different from common neurodegenerative disorders. To understand neuronal changes associated with neurodegeneration, we examined the intrinsic excitability of pyramidal neurons in the CA3b region in the presence of the AMPAR blocker NBQX (10 μM) and the GABA_A_R blocker picrotoxin (50 μM). When we measured membrane potentials by whole-cell current clamp recording, there was no difference in the resting membrane potentials of CA3b pyramidal neurons between genotypes (+/+, − 77.35 ± 1.30 mV; −/−, − 74.60 ± 1.72 mV; t_(22)_ =  − 1.273, *p* = 0.22 by Student’s *t*-test). However, the amplitude of the action potential (AP) elicited by the short (3–5 ms) depolarizing pulse was significantly lower in Xpnpep1^**−/−**^ CA3b neurons than in the WT neurons, while action potential duration (2.63 ± 0.07 vs. 2.55 ± 0.18 ms, t_(22)_ = 0.411, *p* = 0.69 by Student’s *t*-test) was similar in the two genotypes (Fig. 7a,b). The phase-plane (dV/dt) trajectories of APs recorded from Xpnpep1^**−/−**^ CA3 neurons show apparent reduction of dV/dt in the overshoot phase (> 0 mV) and peak, whereas the AP threshold (− 41.48 ± 0.86 vs. − 41.46 ± 1.39 mV, t_(22)_ =  − 0.014, *p* = 0.98 by Student’s *t*-test) and dV/dt during the initial depolarization and repolarization phases did not change (Fig. 7c).

We next determined the passive membrane properties of CA3b pyramidal neurons by measuring the voltage response to a hyperpolarizing current pulse. Unexpectedly, Xpnpep1^**−/−**^ CA3b neurons exhibited more hyperpolarized potentials than Xpnpep1^**+/+**^ neurons in response to the same current (**− **100 pA) injection (Fig. 7d). The values of input resistance (R_in_) for control CA3b neurons were similar to a previous observation^34^, whereas those of Xpnpep1^**−/−**^ CA3b neurons were significantly increased (Fig. 7e). In line with this observation, the membrane time constant (τ_m_) was significantly greater in Xpnpep1^**−/−**^ CA3b neurons than in the WT neurons (Fig. 7f,g). However, the voltage sag ratio, indicative of the activation of hyperpolarization-activated cyclic nucleotide-gated (HCN) channels, in response to the hyperpolarizing current step, did not differ between Xpnpep1^**+/+**^ and Xpnpep1^**−/−**^ CA3b neurons, ruling out the reduction of the HCN current.

Because a hallmark of CA3 pyramidal neurons is burst firing^35,36^, we examined repetitive firing of APs in response to long (500 ms) depolarizing current injections. The firing frequency increased with the intensity of the injected current in both genotypes (Fig. 7h). However, the same current injection produced more spikes in Xpnpep1^**−/−**^ CA3b neurons than WT neurons, and the relationship between the firing frequency and input current (F–I curve) shifted upward in the Xpnpep1^**−/−**^ CA3b neurons (Fig. 7i). The number of spikes elicited by current steps of 300, 400, and 500 pA were significantly different between the two genotypes. When the firing frequencies were fitted with linear regression between 100 and 300 pA of input current, the gain (slope) of the F–I curve was significantly higher in Xpnpep1^**−/−**^ CA3b neurons (0.061 ± 0.007 vs. 0.086 ± 0.008, t_(22)_ =  − 3.397, *p* = 0.025 by Student’s *t*-test). In addition, there was a significant correlation (r^2^ = 0.525, *p* = 0.008) between the slope of the F–I curve and R_in_ of CA3 neurons (Fig. 7j). These results suggest that the increased R_in_ enhances the gain of the input–output relationship and spike firing in Xpnpep1^**−/−**^ CA3 neurons. Previous studies have shown that hippocampal CA3 pyramidal neurons receive frequent and large spontaneous excitatory synaptic transmissions at mossy fiber-CA3 synapses and associational/commissural-CA3 synapses^37,38^. When we measured miniature excitatory postsynaptic currents (mEPSCs) in the principal neurons in the DG, CA1, and CA3 subfields of WT mice, both the amplitude and frequency of mEPSCs were significantly higher in CA3b pyramidal neurons compared with CA1 and DG principal cells (Supplementary Fig. 8). These results indicate that enhanced neuronal excitability in the Xpnpep1^–/–^ CA3 pyramidal neurons and vigorous synaptic excitation at mossy fiber-CA3 synapses and associational/commissural-CA3 synapses may lead to selective excitotoxic cell death of CA3 neurons in the Xpnpep1^–/–^ hippocampus.

Because the R_in_ of a neuron reflects resting membrane conductance, we measured the membrane current elicited by voltage steps (20 mV increments) between − 120 and − 20 mV from a holding potential of − 70 mV (Fig. 7k). Xpnpep1^**−/−**^ CA3b neurons exhibited smaller membrane currents in response to hyperpolarizing (**− **120 to − 80 mV) and near-rest depolarizing (**− **60 mV) voltage steps, while the magnitudes of outward currents induced by higher depolarization (**− **40 and − 20 mV) were not significantly different in CA3b neurons from both genotypes (Fig. 7l). This result indicates that reduction of channels contributing to the resting conductance is responsible for the enhanced R_in_ of CA3 neurons in the Xpnpep1^**−/−**^ mice.

## Discussion

Although inborn errors of metabolism (IEMs) are common causes of brain dysfunction and intellectual disability, the molecular and cellular changes associated with brain dysfunction are unknown in most metabolic diseases. The present study demonstrates previously unknown changes in the hippocampus with inherited metabolic disease in which long-term exposure of the brain to the altered metabolic status in aminopeptidase P1 deficiency modifies hippocampal gene expression, glial population, and neuronal excitability in CA3 neurons. Specifically, the density of microglia in the hippocampal CA3 subfield was higher in Xpnpep1^**−/−**^ mice than in WT mice, whereas the mutant mice exhibited fewer astrocytes in the hippocampus. Interestingly, astrocyte activation or reactive gliosis was not detected in the Xpnpep1^**−/−**^ hippocampus. Xpnpep1^**−/−**^ CA3 pyramidal neurons exhibited enhanced gain in the input–output relationship, such that the same current injection produced more spikes in Xpnpep1^**−/−**^ neurons than in WT neurons. Thus, an aberrant glial environment and enhanced excitability in CA3 neurons might cause neurodegeneration and hippocampal dysfunction in aminopeptidase P1 deficiency.

As IEMs are usually caused by defects in enzymes, accumulation of substrates for the defective enzyme in the cerebrospinal fluid is considered to cause brain dysfunction and injury, but long-term changes in the brains exposed to the accumulated substrates are unclear. In the present study, we identified 14 down-regulated genes in the Xpnpep1^**−/−**^ hippocampus through microarray and qRT-PCR. There is a possibility that some down-regulated genes that are abundant in astrocytes might be identified as DEGs due to the reduction of astrocyte density in the Xpnpep1^**−/−**^ hippocampus. Intriguingly, a previous report showed that Slc6a20 mRNA was mainly detected in microglia, meninges, and choroid plexuses in the brain^12^. Although Xpnpep1^**−/−**^ mice exhibit an increased number of microglia in the hippocampal CA3 subfields, the expression levels of Slc6a20a transcripts in the hippocampus were decreased. Slc6a20a transports imino acids, including proline and hydroxy proline, through the Na^+^- and Cl^–^-dependent mechanisms^12,13^. The reduction of Slc6a20a in the Xpnpep1^**−/−**^ hippocampus probably indicates an altered imino acid gradient across the plasma membrane of brain cells and further suggests adaptive changes in the Xpnpep1^**−/−**^ brain. Similarly, the reduced mRNA expression of Slc22a2 (OCT2), a transporter for norepinephrine (NE) and serotonin (5-HT), seems to be associated with adaptive changes in the Xpnpep1^**−/−**^ brain. Slc22a2 is highly expressed in limbic neurons including CA1 and CA3 pyramidal neurons, but not in astrocytes in the brain, and contributes to the clearance of NE and 5-HT, which suppress the firing activity of CA3 pyramidal neurons^39^. Reduction of Slc22a2 may slow the clearance of NE and 5-HT, thereby intensifying the suppression of abnormally excitable CA3 neurons in Xpnpep1^**−/−**^ mice. Another interesting finding from the gene expression profile of the Xpnpep1^**−/−**^ hippocampus was the down-regulation of desmoplakin (Dsp). Dsp is exclusively expressed in dentate granule cells (GCs) in the hippocampus, and the expression level of Dsp increases with postnatal development^14,22^. Considering that reduced Dsp expression is a sign of the “immature dentate gyrus (iDG)”, which is frequently observed in genetically engineered mice with abnormal behaviors^40^, our results suggest that neurodegeneration and delayed neurodevelopment coincide in aminopeptidase P1 deficiency^41^. Intriguingly, mice with the iDG phenotype exhibit hyperexcitability of the dentate GCs. The existence of developmental retardation, hyperlocomotion, and impaired hippocampus-dependent learning in Xpnpep1^**−/−**^ mice^7,8^ means it is likely that iDG, in addition to the hyperexcitable CA3 pyramidal neurons, contributes to hippocampal dysfunction. However, the excitability of Xpnpep1^**−/−**^ GCs requires further investigation.

Although Xpnpep1^**−/−**^ mice exhibit neurodegenerative cell death and vacuolation in the hippocampal CA3 region, glial alterations in the Xpnpep1^**−/−**^ hippocampus were quite different from those observed in common neurodegenerative diseases in that the density of astrocytes and expression levels of GFAP were reduced in the Xpnpep1^**−/−**^ hippocampus, while the density of microglia was increased specifically in the CA3 subfields. Intriguingly, microglial activation was not observed in the Xpnpep1^**−/−**^ mice. This observation suggests that the increased number of microglia in the Xpnpep1^**−/−**^ CA3 area might be associated with physiological housekeeping functions, such as migration to sites of neuronal death to phagocytose cellular debris or apoptotic cells, rather than initiation or exacerbation of neurodegeneration^33^.

Notably, consistent with the previous in situ hybridization results (Supplementary Fig. 2)^42^, we observed predominant Xpnpep1-expression in neurons, but relatively weak expression in glial cells (Fig. 3 and Supplementary Fig. 4). These observations indicate that the substrates of aminopeptidase P1 in the hippocampus are mostly cleared within the neurons and that accumulation of undigested peptide substrates containing a penultimate proline residue in the cerebrospinal fluid influences the glial population and neuronal excitability. Considering that a variety of oligopeptides with N-terminal X-pro motifs exhibit diverse biological activities^10^, long-term exposure of brain cells to the substrates of aminopeptidase P1 seems to result in altered gene expression in brain cells and neuro-glial interactions.

Activation of astrocytes with an increased expression of GFAP and S100β is a hallmark of brain diseases, including common late-onset neurodegenerative diseases, ischemic brain injuries, and epilepsy^27,30,43,44^. Moreover, phenylketonuria and homocystinuria, inborn errors of metabolism caused by the deficiency of phenylalanine hydroxylase and cystathionine β-synthase respectively, also exhibit gliosis in the brain^45,46^. Meanwhile, chronic unpredictable stress and starvation induced a reduction in astrocytes in the cerebral cortex^31,47^. A recent study showed that depletion of astrocytes by treatment with the gliotoxin L-α-aminoadipic acid (L-α-AAA) did not induce neuronal death in the hippocampal CA3 area without insults such as ischemia, but that the loss of astrocyte produced persistent Ca^2+^ increase in the CA3 neurons after ischemia and reperfusion^48^. Thus, the reduction of astrocytes in the Xpnpep1^**−/−**^ hippocampus likely results in insufficient neuroprotection against intracellular Ca^2+^ load during burst firing or the hyperactivation of neurons rather than direct induction of neurodegeneration in the Xpnpep1^**−/−**^ CA3 subfield. It has been suggested that S100β, an astrocytic calcium-binding protein, protects neurons against NMDA-induced cell death by activating nuclear factor κB (NF-κB) signaling^49^. In addition to neuroprotection, S100β released from astrocytes regulates neuronal activity and oscillations^50,51^. Therefore, fewer astrocytes and resultant insufficient S100β release seem to be associated with hippocampal dysfunction in Xpnpep1^**−/−**^ mice.

In the present study, we found that the excitability and AP waveform of CA3b neurons were changed by aminopeptidase P1 deficiency. The peak amplitudes of APs and the derivative (dV/dt) of membrane potentials during the overshoot phase were significantly reduced in the Xpnpep1^**−/−**^ CA3 neurons, whereas disruption of Xpnpep1 had no effect on other AP parameters including resting membrane potentials, AP threshold, AP duration, and dV/dt during the initial depolarization and repolarization phases. The reduction of AP peak amplitudes and dV/dt during the overshoot phase is likely caused by alterations in Na^+^ currents but not by increased I_A_ (A-type K^+^ channel current) or I_D_ (D-type K^+^ channel current) because blockade of I_A_ and I_D_ slowed the repolarization of CA3 neurons without affecting the peak amplitude of AP^52,53^. Despite the reduced AP amplitudes, the same current injection produced more spikes in Xpnpep1^**−/−**^ CA3b neurons than WT neurons because of the increased input resistance (R_in_). The enhanced excitability with reduced AP amplitude resembles oxytocinergic modulation of intrinsic properties of CA2 pyramidal neurons, in which reduction of AP amplitude induced by the activation of oxytocin receptor was blocked by intracellular calcium chelation^54^. In hippocampal CA3 pyramidal neurons, Na_V_1.2 is distributed to the soma and along the axon evenly, while Na_V_1.6 is concentrated to the distal axon initial segment (AIS)^55^. Based on the subcellular localization, Na_V_1.2 is considered to play a critical role in AP propagation and somatic AP generation, whereas Na_V_1.6 seems to determine the initiation and threshold of AP^55,56^, indicating that Na_V_1.2 is probably associated with reduced dV/dt during the overshoot phase in Xpnpep1^**−/−**^ CA3 neurons. Interestingly, haploinsufficiency of Na_V_1.2 in excitatory but not inhibitory neurons resulted in absence-like seizures with epileptiform electrocorticography (ECoG) activities in mice^57^. This finding implies that reduction of Na^+^ channel expression does not necessarily reduce excitability in neurons, but that reduced function of certain isoforms of Na^+^ channels can enhance neuronal activity. However, it should be noted that ionic mechanisms enhancing intrinsic excitability and reducing the amplitude of AP in Xpnpep1^**−/−**^ CA3 neurons are dissociable. We observed that the slope (gain) of firing frequency (output)-the injected current (input) correlated well with the R_in_ of CA3b neurons (Fig. 7). This observation indicates that altered R_in_ resulting from the decreased resting conductance is mainly responsible for enhanced AP firing in Xpnpep1^**−/−**^ CA3 neurons. As spontaneous excitatory synaptic inputs onto CA3 neurons are more frequent and larger than DG or CA1 neurons (Supplementary Fig. 8), it is conceivable that vigorous spike firing by enhanced excitability and robust synaptic excitation result in selective neurodegeneration of CA3 neurons in Xpnpep1^**−/−**^ mice, despite the fact that they have fewer astrocytes in the entire hippocampus. Although the ion channels responsible for reduced resting conductance in Xpnpep1^**−/−**^ CA3 neurons require further identification, the present study provides cellular mechanisms underlying hippocampal dysfunction and CA3 neurodegeneration in aminopeptidase P1 deficiency.

## Methods

### Animals

Generation and genotyping of Xpnpep1 knockout mice has been previously described^7^. Mice were backcrossed to C57BL/6J and 129S4/SvJae mice for at least 10 generations before use. All analyses were performed on littermates of both genotypes generated by intercrosses between C57BL/6J and 129S4/SvJae heterozygous parents. Animals were housed 4–5 per cage in a specific pathogen-free facility, and maintained in a climate-controlled room with free access to food and water in a 12 h light/dark cycle with the light on at 7:00. Animal maintenance and all animal experiments were performed under protocols approved by the Institutional Animal Care and Use Committee (IACUC) at Seoul National University. All methods were carried out in accordance with Guidelines for the Care and Use of Mammals in Neuroscience and Behavioral Research (National Research Council, US).

### Microarray and qRT-PCR

Hippocampi were removed from 4- to 5-week-old mice of both sexes and incubated in RNA stabilization reagent (RNAlater, Qiagen, USA) at 4 °C overnight. Total hippocampal RNA was prepared using QIAzol reagent and then cleaned using the RNeasy Mini Kit (Qiagen, USA) according to the manufacturer’s protocol. To evaluate the integrity of the prepared RNA samples, the RNA integrity number (RIN) was determined using an Agilent 2100 Bioanalyzer (Agilent Technologies, USA), and samples with RIN (+/+, 7.37 ± 0.025; −/−, 7.25 ± 0.095) higher than 7.1 were used for further processing. The purity of RNA samples was evaluated using a spectrophotometer (NanoDrop ND-1000, Thermo Fisher Scientific, USA) by measuring the A260/A280 ratio (+/+, 2.057 ± 0.029; −/−, 2.06 ± 0.012) and the A260/A230 ratio (+/+, 1.93 ± 0.14; −/−, 1.92 ± 0.21).

Synthesis of first- and second-strand (ss) cDNA, cRNA amplification and purification, second cycle synthesis of ss-cDNA and purification, fragmentation of ss-cDNA, and biotinylation of the fragmented cDNA were conducted according to the Affymetrix GeneChip procedure. The labeled fragmented cDNA was hybridized to the microarray chip (GeneChip Mouse Gene 1.0 ST array, Affymetrix, USA) containing 28,853 gene-level probe sets (770,317 distinct probes), and the hybridized probe array was stained with streptavidin-coupled fluorescent dye. The stained arrays were scanned with an Affymetrix GeneChip 3000 scanner, and the signal intensity of the gene expression levels was determined using Affymetrix Expression Console software. Hierarchical clustering and heatmap generation were performed using Morpheus software (https://software.broadinstitute.org/morpheus/).

For qRT-PCR, first-strand cDNAs were synthesized from the total RNA with oligo (dT) primer using AMV reverse transcriptase (NEB, MA, USA) at 37 °C for 1 h. The cDNA templates were mixed with forward and reverse primers (Supplementary Table 1) and IQ SYBR Green Supermix (Bio-Rad, CA, USA). The real-time PCR analyses were performed using the CFX Connect Real-Time PCR detection system (Bio-Rad) using the following thermal cycling protocol: initial denaturation for 10 min at 95 °C; and 40 cycles alternating 15 s at 95 °C and 1 min at melting temperature. Melting curves and data analyses were performed with Precision Melt Analysis Software and CFX Manager software (Bio-Rad). The housekeeping gene Gapdh was used as a reference gene. The specificity and efficiency of all primer pairs were confirmed by RT-PCR and agarose gel electrophoresis (Supplementary Fig. 9).

### Primary neuron culture and transfection

Hippocampi were collected from embryonic day 18–19 rats and were incubated in HBSS containing 2.5% trypsin at 37 °C for 20 min. After rinsing with HBSS 3 times, neurons were dissociated by repeated trituration with a fire-polished Pasteur pipet, and were plated on coverslips coated with poly-l-lysine and laminin. Neurons were cultured in neurobasal medium supplemented with B27 (Invitrogen), 2 mM l-glutamine, 1% (v/v) penicillin/streptomycin (100 U/ml, Gibco), and 2% fetal bovine serum (Gibco) in a 10% CO_2_ incubator. For the IRES-mediated expression of epitope (HA or Flag)-tagged aminopeptidase P1 in cultured neurons, the eGFP sequence was PCR amplified from the pEGFP-N1 vector and inserted into the pGW1 vector at the HindIII and KpnI sites. The vector was serially digested with KpnI, Mung bean nuclease, and BglII to yield upstream blunt and downstream sticky ends. The IRES sequence was PCR-amplified from the pIRES2-EGFP vector using 5′ phosphorylated forward primer and the reverse primer containing the recognition sequences for BglII, digested with BglII, and then ligated to the digested pGW1-eGFP vector. In parallel, Xpnpep1 cDNA was amplified from rat hippocampal cDNA using PCR primers containing the SalI recognition sequence (forward primer) and FLAG- or HA-EcoRI sequences (reverse primer). Epitope (HA or FLAG)-tagged Xpnpep1 was inserted into the pGW1-eGFP-IRES vector at the SalI and EcoRI sites. The sequence-verified constructs were transfected into cultured hippocampal neurons at DIV 12 using a calcium phosphate transfection kit (Takara Bio Inc. and Promega), and neurons were fixed with 4% paraformaldehyde/4% sucrose at DIV 14.

Glia-free hippocampal neuron cultures and neuron-free glial cultures were prepared from embryonic day 18–19 mice according to the protocol described above. To establish glia-free neuronal culture, dissociated hippocampal cells were cultivated in serum-free neurobasal medium, and cells were treated with the antimitotic agent AraC (3 μM; Sigma) for 8 days from DIV 12. AraC was then removed from the culture by washing the cells with fresh neuron culture medium, and neurons were harvested at DIV 21. To obtain neuron-free glial cultures, dissociated hippocampal cells were cultured in Dulbecco’s modified Eagle’s medium containing 2.5 mM glucose, 4 mM l-glutamine, 3.7 g/L sodium bicarbonate, 10% (v/v) FBS, 1 mM sodium pyruvate, and 1% (v/v) penicillin/streptomycin. The cell culture medium was replaced with fresh media once every 3 days. To remove neurons, cells were detached with 0.25% trypsin-EDTA at DIV 9 and plated in a new culture dish. The cells were harvested at DIV 19 when they reached 90–100% confluence.

### X-gal staining and immunofluorescence staining

Four- to five-week-old male mice were deeply anesthetized with a mixture of Zoletil (50 mg/kg, intraperitoneally [i.p.]) and xylazine (1 mg/kg, i.p), and transcardially perfused with phosphate-buffered saline (PBS), followed by treatment with a fixative containing 4% (w/v) paraformaldehyde in PBS. Mouse brains were removed, post-fixed for 12 h at 4 °C, and cut into 100 μm-thick sections using a vibratome (VT1200S, Leica, Germany). The sections were incubated in X-gal staining solution (5 mM K_3_Fe(CN)_6_, 5 mM K_4_Fe(CN)_6_, 2 mM MgCl_2_, 0.01% deoxycholate, 0.02% NP-40, and 1 mg/mL X-gal in PBS) at 37 °C for 5–8 h and then post-fixed for 1 h at 4 °C. The sections were rinsed 3 times for 10 min with PBS and stored at 4 °C until use. Light microscope images were acquired using a microscope (BX51WI, Olympus, Japan) equipped with a cooled charge-coupled device camera (DP73, Olympus, Japan).

For immunofluorescence staining, formalin-fixed hippocampal sections or cultured hippocampal neurons were permeabilized with 0.3% (v/v) Triton X-100 in PBS and incubated in a blocking buffer (5% normal goat serum, 5% horse serum, 5% donkey serum, and 0.5% BSA in PBS) for 2 h. Samples were successively incubated with primary antibodies (anti-FLAG: Sigma-Aldrich, Cat. # F1804; anti-HA: Santa Cruz Biotechnology, Cat. # sc-805; anti-GFP: Synaptic Systems, Cat. # 132 004; anti-NeuN: Millipore, Cat. # ABN78; anti-MAP2: Sigma-Aldrich, Cat. # M9942; anti-GFAP: Abcam, Cat. # ab7260, Sigma-Aldrich, Cat. # G3893; anti-S100β: Sigma-Aldrich, Cat. # S2532; anti-CD68: Bio-Rad, Cat. # MCA1957GA; anti-Iba1: Novus Biologicals, Cat. # NB100-1028; anti-O4: Millipore, Cat. # MAB345; overnight at 4 °C) and fluorescence (FITC, Cy3, or Alexa Fluor 647: Jackson ImmunoResearch Laboratories, PA, USA) conjugated secondary (3 h at room temperature) antibodies. After each step, the samples were rinsed 3 times for 10 min with PBS. Stained sections or neurons were mounted on a glass slide using a fluorescence mounting medium containing DAPI (Abcam, Cat. # ab104139). Images (12 bit, 2048 × 2048 pixel resolution) were acquired (pixel dwell of 4 µs, line average of 3) using a confocal laser scanning microscope (FV 3000, Olympus, Japan). Fluorescence signals were visualized using 405 nm (50 mW), 488 nm (20 mW), 561 nm (20 mW), and 640 (40 mW) lasers. Laser power and voltage of photomultiplier tube (PMT) were set to 1–5% of the maximal output and 350–500 V, respectively.

To measure the cross-sectional area of the hippocampus, light microscope images of the hippocampus from brain sections were acquired using a microscope (BX51WI, Olympus, Japan), and images were analyzed using MetaMorph software (Molecular Devices, Sunnyvale, USA).

### Western blotting

Hippocampal lysates were prepared from 4- to 5-week-old mice of both sexes. Hippocampi were homogenized in a homogenization buffer (320 mM sucrose, 10 mM Tris-HCl, 5 mM EDTA, pH 7.4) containing protease inhibitor cocktails (Sigma, P8340) and phosphatase inhibitor cocktail (GenDepot, P3200). Subcellular brain fractions were prepared as described^58^. Briefly, rat brain homogenates (H) were centrifuged at 1000×*g* to remove nuclei and other large debris (P1). The supernatant (S1) was centrifuged at 10,000×*g* to obtain a crude synaptosomal fraction (P2), and the supernatant (S2) was further centrifuged at 160,000×*g* to obtain light membranes (P3) and cytosolic fractions (S3). The P2 fraction was subjected to hypotonic lysis and centrifuged at 25,000×*g* to precipitate synaptosomal membranes (LP1).

To extract proteins from cultured hippocampal neurons or glial cells, cells were washed twice with chilled PBS and subsequently treated with a cell lysis buffer containing 1% Triton X-100, protease inhibitor cocktails, and phosphatase inhibitor cocktails. Cell lysates were centrifuged at 10,000×*g* at 4 °C for 15 min and supernatants were collected.

The total protein concentrations of the hippocampal homogenates, cell lysates, or subcellular brain fractions were determined using the Bradford protein assay (Bio-Rad, Hercules, USA). Proteins were dissolved and boiled for 5 min in an SDS sample loading buffer (pH 6.8) containing 50 mM Tris-HCl, 2% (w/v) SDS, 10% (v/v) glycerol, 12.5 mM EDTA, 0.02% (w/v) bromophenol blue, and 5% (v/v) 2-mercaptoethanol. Samples containing 10–15 μg of protein were loaded onto SDS-PAGE gels. The separated proteins were transferred to nitrocellulose membranes. The membranes were blocked in Tris-buffered saline (TBST, 0.1% Tween 20) containing 5% skim milk for 30 min at room temperature, and then successively incubated with diluted primary and horseradish peroxidase (HRP)-conjugated secondary antibodies for 1 h at room temperature. After each step, the membranes were rinsed 3 times for 10 min with TBST. The HRP signals were detected by enhanced chemiluminescence (GE Healthcare, UK). The production of polyclonal aminopeptidase P1-antibody has been described previously^7^. Anti-α-tubulin (Cat. # T5168) and anti-βIII-tubulin (Cat. # T8660) antibodies were purchased from Sigma-Aldrich (USA). All western blot experiments were independently repeated at least 3 times, and band intensities were quantified using MetaMorph software (Molecular Devices, Sunnyvale, USA).

### Electrophysiology

Electrophysiological recordings from hippocampal slices were performed as described previously^59^. Briefly, hippocampal slices (400 μm) were prepared from 4- to 5-week-old male mice using a vibratome (VT1000S; Leica, Germany) in ice cold dissection buffer (in mM: sucrose 213, NaHCO_3_ 26, KCl 2.5, NaH_2_PO_4_ 1.25, d-glucose 10, Na-pyruvate 2, Na-ascorbate 1.3, MgCl_2_ 3.5, and CaCl_2_ 0.5 bubbled with 95% O_2_/5% CO_2_). The slices were recovered at 36 °C for 1 h in the artificial cerebrospinal fluid (aCSF; in mM: NaCl 125, NaHCO_3_ 26, KCl 2.5, NaH_2_PO_4_ 1.25, d-glucose 10, MgCl_2_ 1.3, and CaCl_2_ 2.5), followed by maintenance at room temperature until use. Individual slices were then transferred to a recording chamber and perfused with aCSF at 30 °C. Whole-cell patch clamp recordings were performed using a MultiClamp 700B amplifier (Molecular Devices, USA). Signals were low-pass filtered at 2.8 kHz and digitized at 10 kHz using a Digidata 1440A digitizer (Molecular Devices, USA). Membrane potentials or currents of CA3b neurons were recorded using a pipette (3–4 MΩ) solution containing (in mM) K-gluconate 110, KCl 20, NaCl 8, HEPES 10, Mg-ATP 4, Na-GTP 0.3, and EGTA 0.5 (with pH 7.25, 290 mOsm). During the recording, the GABA_A_R blocker picrotoxin (50 μM), an APMAR blocker NBQX (10 μM), and an NMDAR blocker AP-5 (50 μM) were included in the aCSF. Recordings were started 10 min after establishment of the whole-cell configuration. The series resistance (< 10 MΩ) and seal resistance (> 1 GΩ) were monitored before and after recordings by applying a short (50 ms) hyperpolarization voltage pulse (− 5 mV), and the data were discarded if the resistance changed by more than 20% during the recording. In the current clamp experiments, neurons displaying an unstable resting membrane potential (RMP) at the beginning or during the recording were discarded. Action potentials (APs) were evoked by a brief (2–3 ms) minimal current (0.6–1 nA) injection. The amplitude of AP was measured as the difference between the peak voltage of spike and the baseline voltage (RMP). AP threshold was defined as the membrane potential at the clear inflection point between the electrotonic potential and the AP. AP duration was measured from threshold to 90% repolarization. Miniature excitatory postsynaptic currents (mEPSCs) were recorded at the holding potential of − 60 mV in the hippocampal principal cells in the presence of the GABA_A_R blocker picrotoxin (50 µM) and tetrodotoxin (1 µM) in the aCSF. All data were analyzed using custom macros written in Igor Pro 6 (WaveMetrics, OR, USA). All chemicals were purchased from Sigma-Aldrich (USA), except for picrotoxin, NBQX, and AP-5 (Tocris, UK).

### Statistical analysis

Statistical analyses were performed using SPSS software (IBM Corporation, NY, USA). The Shapiro–Wilk test was used to assess normality of the collected data. The two-tailed Student’s *t*-test and the Mann–Whitney test were used to compare normally distributed and non-normally distributed samples, respectively. The one-way analysis of variance (ANOVA) with Tukey’s post-hoc test was used for three groups of data. The levels of statistical significance are indicated as follows: n.s., not significant (*p* ≥ 0.05), *p < 0.05, **p < 0.01, ***p < 0.001. All bar and line graphs in the figures are presented as the mean ± standard error of mean (SEM).

## Supplementary Information

Supplementary information.
