# Supplementary material for: Altered hippocampal gene expression, glial cell population, and neuronal excitability in aminopeptidase P1 deficiency
Source: Sci Rep. 2021 Jan 13;11:932. doi: 10.1038/s41598-020-79656-6 (PMC7806765; doi:10.1038/s41598-020-79656-6)
Supplement: Supplementary file 1 — Supplementary information. [file 41598_2020_79656_MOESM1_ESM.pdf]

Supplementary Figures and Table

Altered hippocampal gene expression, glial cell population, and neuronal excitability in aminopeptidase P1 deficiency

Sang Ho Yoon,[1,2] Young-Soo Bae,[1] Sung Pyo Oh,[1] Woo Seok Song,[1,2] Hanna Chang,[1] and Myoung-Hwan Kim[1,2,3]

[1]Department of Physiology and Biomedical Sciences, Seoul National University College of Medicine, Seoul, 03080, Korea. [2]Neuroscience Research Institute, Seoul National University Medical Research Center, Seoul, 03080, Korea. [3]Seoul National University Bundang Hospital, Seongnam, Gyeonggi, 13620, Korea.

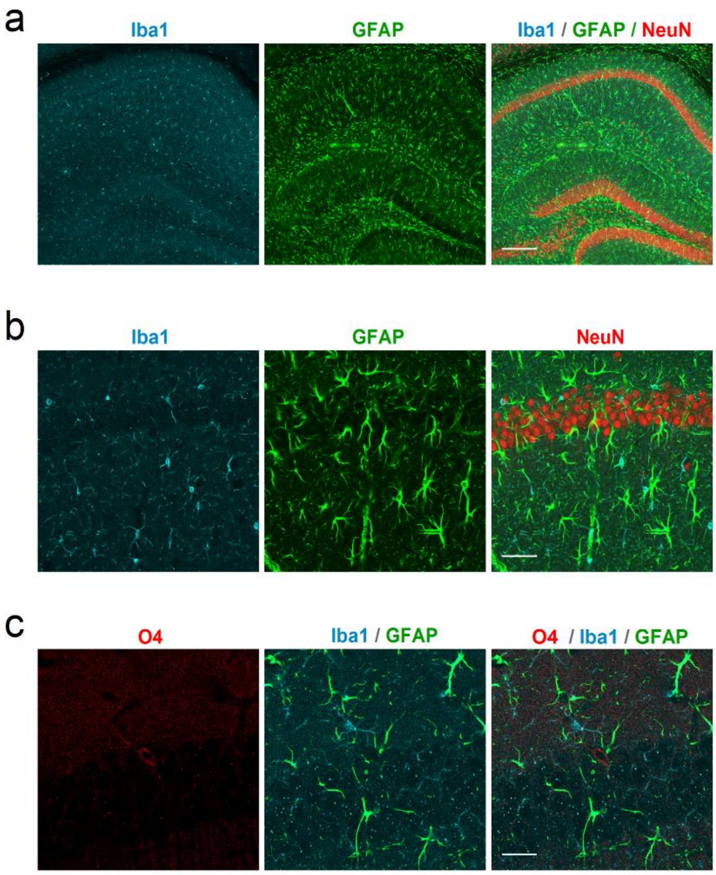

Supplementary Figure 1. Immunohistochemical labeling of neurons and glial cells in the mouse hippocampus. (a) Iba1-positive signals were evenly distributed throughout the hippocampus (left). GFAP-immunoreactive signals were enriched in the CA1 stratum lacunosum-moleculare and DG hilus, while principal cell layers exhibit relatively low density of GFAP-positive cells (middle). NeuN-immunoreactive signals were mostly detected in the principal cell layers (right). Scale bar, 200 μm. (b) Higher magnification images show that each antibody labels distinct cell types within the hippocampus. Scale bar, 50 μm. (c) Iba1, GFAP, and O4-postive cells constitute different populations of glial cells in the hippocampus. Scale bar, 25 μm.

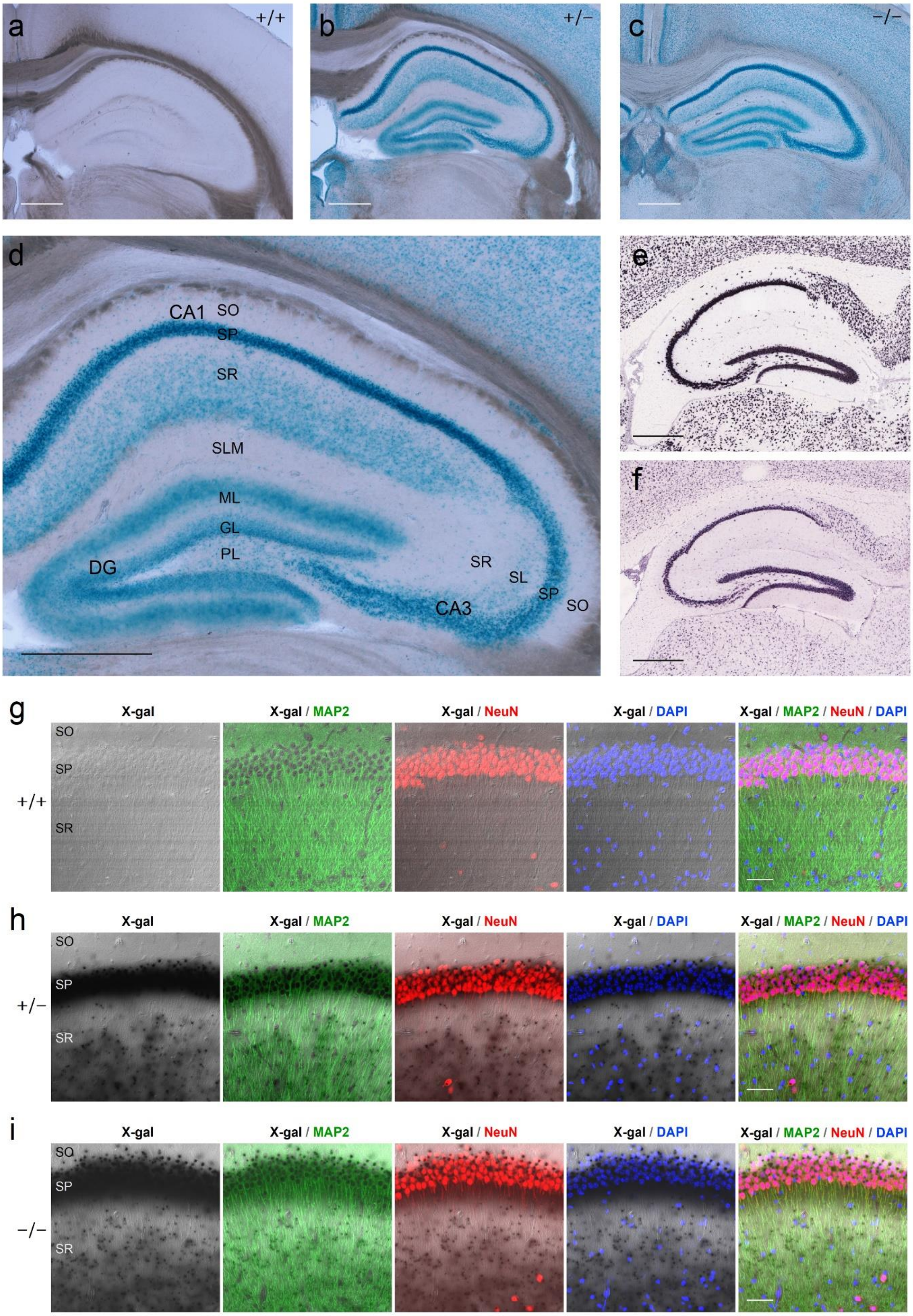
a
+/+
b
+/−
c
−/−
d
CA1
SO
SP
SR
SLM
ML
GL
PL
DG
SR
SL
SP
SO
CA3
e
f
g
X-gal
X-gal / MAP2
X-gal / NeuN
X-gal / DAPI
X-gal / MAP2 / NeuN / DAPI
SO
SP
SR
+/+
h
+/−
i
−/−

Supplementary Figure 2. Aminopeptidase P1 is abundantly expressed in neurons. (a) X-gal signals were not detected in the $Xpnpep1^{+/+}$ hippocampus. (b-c) Light-microscope images showing the X-gal staining pattern in the $Xpnpep1^{+/-}$ (b) and $Xpnpep1^{-/-}$ (c) hippocampi. (d) Higher magnification view of $Xpnpep1^{+/-}$ hippocampus shown in (b). *Stratum pyramidale* (SP) exhibits the strongest X-gal signal and disperse X-gal precipitates were detected in the *stratum radiatum* (SR) of the $Xpnpep1^{+/-}$ CA1 subfield. Occasional punctate X-gal signals were detected in the CA1 *stratum oriens* (SO) and *stratum lacunosum-moleculare* (SLM). X-gal inclusions were observed in the outer molecular layer (ML), granule cell layer (GL), and hilus (polymorphic layer, PL) of the DG. Within the CA3 subfield, X-gal signals were mainly detected in the pyramidal layer and *stratum lucidum* (SL). (e) In-situ hybridization image of neuron specific enolase (enolase 2, Eno2) showing distribution of neurons in the hippocampus (Allen Brain Atlas Experiment #:112197625). (f) Distribution of Xpnpep1 mRNA in the hippocampus (Allen Brain Atlas Experiment #:70195054) shows a pattern similar to that of Eno2. Scale bars, 500 μm (a-f). (g-i) X-gal inclusions in the hippocampal CA1 subfield were detected as dark puncta in the $Xpnpep1^{+/-}$ (h) and $Xpnpep1^{-/-}$ (i) but not in the $Xpnpep1^{+/+}$ (g) mice by confocal microscopy with transmitted light illumination (left). Transmitted image was merged with confocal fluorescence images of MAP2, NeuN, and DAPI, respectively (middle). Right, combined transmission and fluorescence of images show the presence of X-gal precipitates in the somata and dendrites of $Xpnpep1^{+/-}$ (h) and $Xpnpep1^{-/-}$ (i) CA1 neurons. Scale bars, 50 μm (g-i).

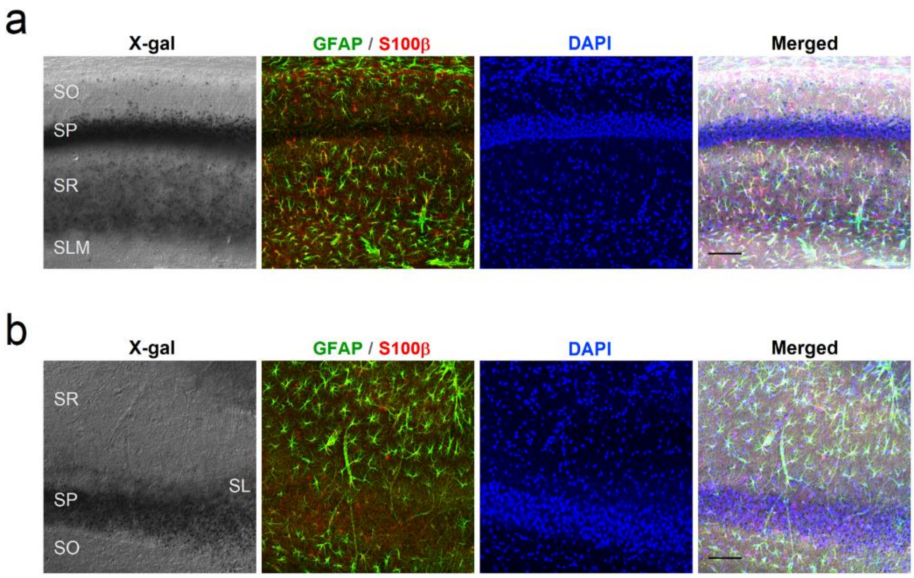

Supplementary Figure 3. Distribution of astrocytes and X-gal precipitates in the Xpnpep1$^{+/-}$ hippocampus. (a) The X-gal stained sections were immunostained with GFAP and S100β to visualize astrocytes in the hippocampus. Cell nuclei were labeled with DAPI. Note the enrichment of astrocytes in the CA1 *stratum oriens* (SO) and *stratum lacunosum-moleculare* (SLM), in which X-gal precipitates were rarely observed. (b) Astrocytes in the CA3 were abundant in the *stratum radium* (SR) and *stratum oriens* (SO), whereas X-gal signals were mostly detected in the *stratum lucidum* (SL) and *stratum pyramidale* (SP). Scale bars, 100 μm (a and b).

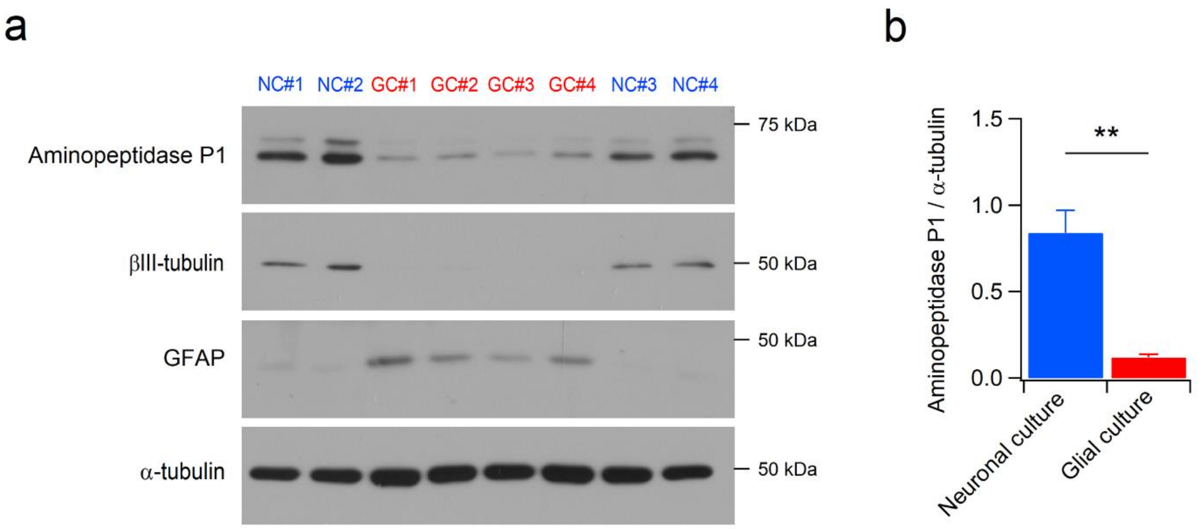

Supplementary Figure 4. Protein expression levels of aminopeptidase P1 were significantly higher in neuronal lysates than in glial lysates. (a) Total proteins extracted from 4 independent batches of cultured hippocampal neurons (NC) and glial cells (GC) were separated by SDS-PAGE. Aminopeptidase P1-immunoreactive signals were much stronger in neuronal lysates than in glial lysates. Immunoreactive signals for GFAP and βIII-tubulin were mainly detected in glial and neuronal lysates, respectively. (b) Quantification of endogenous aminopeptidase P1 protein levels (right) in the neuronal (n = 4) and glial (n = 4) lysates. $t_{(6)} = 5.44$, $^{**}p = 0.0016$ by Student's *t*-test.

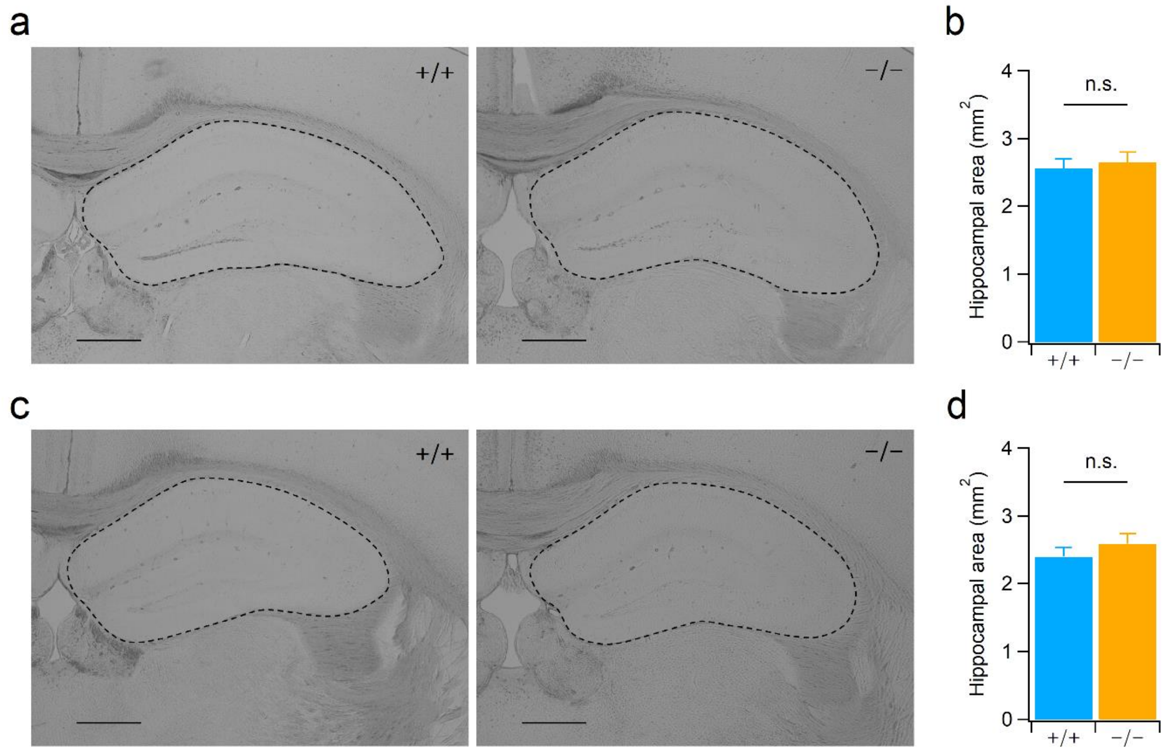

Supplementary Figure 5. The areas of hippocampi in the brain sections used for the analysis of glial cell density. (a) Representative bright-field images of the hippocampus from brain sections of WT (left) and Xpnpep1$^{-/-}$ (right) mice. Hippocampi are marked with a dotted line. (b) The areas of hippocampi in the brain sections used for the immunohistochemical analysis of astrocytes were not different between WT and Xpnpep1$^{-/-}$ (right) mice. n = 6 slices from 3 mice for each genotype; $t_{(10)} = -0.43$; n.s., not significant; p = 0.68 by Student's *t*-test. (c, d) Sample bright-field images (c) and the areas (d) of hippocampi used for the analysis of microglial cells. n = 6 slices from 3 mice for each genotype; $t_{(10)} = -0.96$; n.s., not significant; p = 0.36 by Student's *t*-test. Scale bars, 500 μm (a and c).

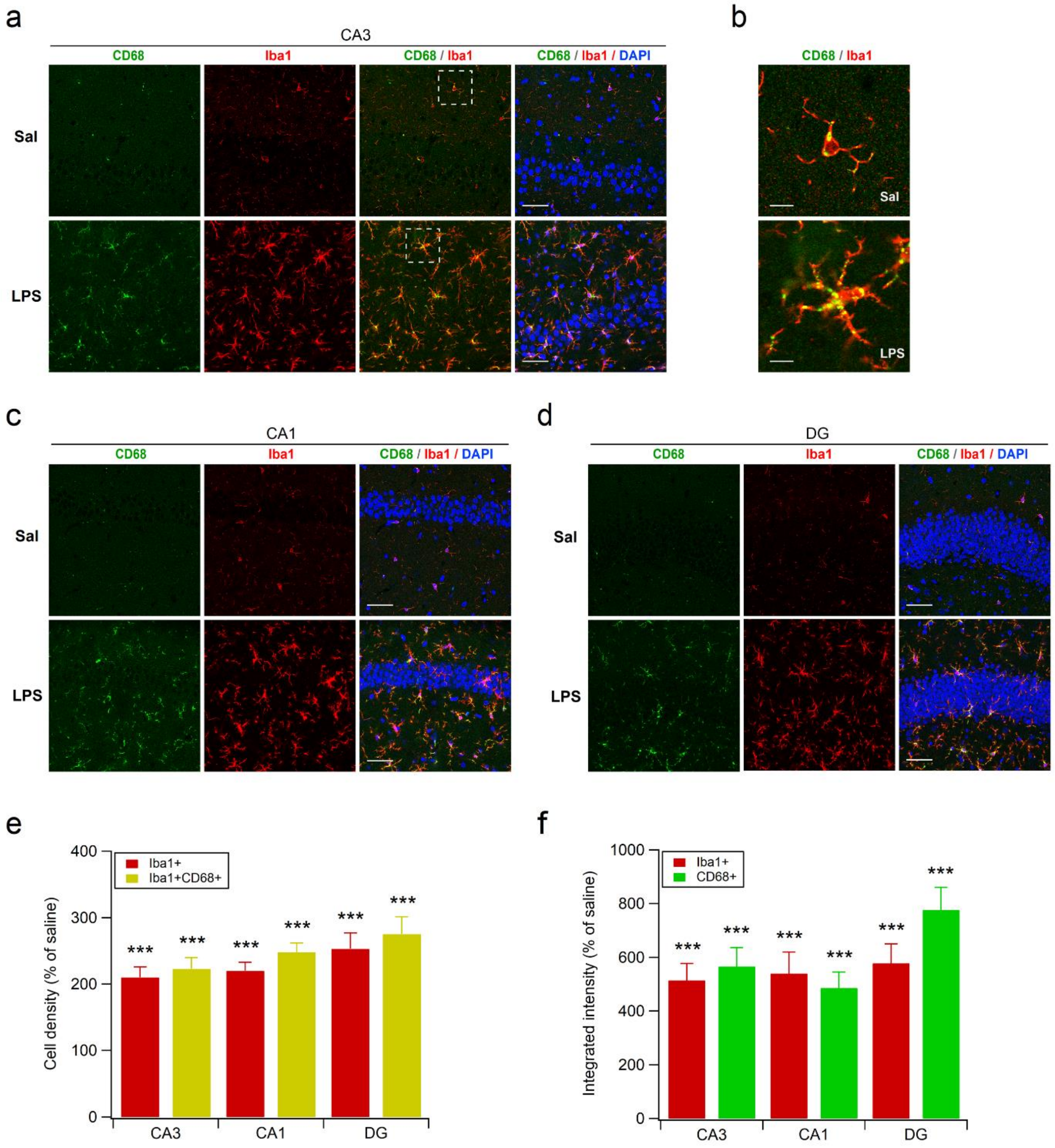

Supplementary Figure 6. Punctate CD68-immunoreactive signals in hippocampal microglia may be indicative of a resting state microglia. (a) LPS treatment (once daily for 4 days) results in an increased density of microglia and enhanced expression of Iba1 and CD68 proteins in the hippocampal CA3 region of Xpnpep1$^{+/+}$ mice. (b) Higher magnification views of areas indicated by the dotted white box in panel (a) show differences in morphology and distribution of CD68-immunoreactive signals between control (top) and LPS-activated (bottom)

microglia. (c, d) Representative fluorescence images of Iba1, CD68, and DAPI showing LPS-induced activation of microglia in the hippocampal CA1 (c) and DG (d) subfields of Xpnpep1$^{+/+}$ mice. Scale bars, 50 μm (a, c, and d) and 10 μm (b). (e, f) Quantification of microglial cell density (e) and fluorescence intensity (f) across the hippocampal subfields in LPS-treated mice. Data are normalized to those of saline-treated mice. n = 12 slices from 6 mice in each group. $t_{(10)} = -4.29 \sim -9.55$, ***$p < 0.001$ by Student's *t*-test.

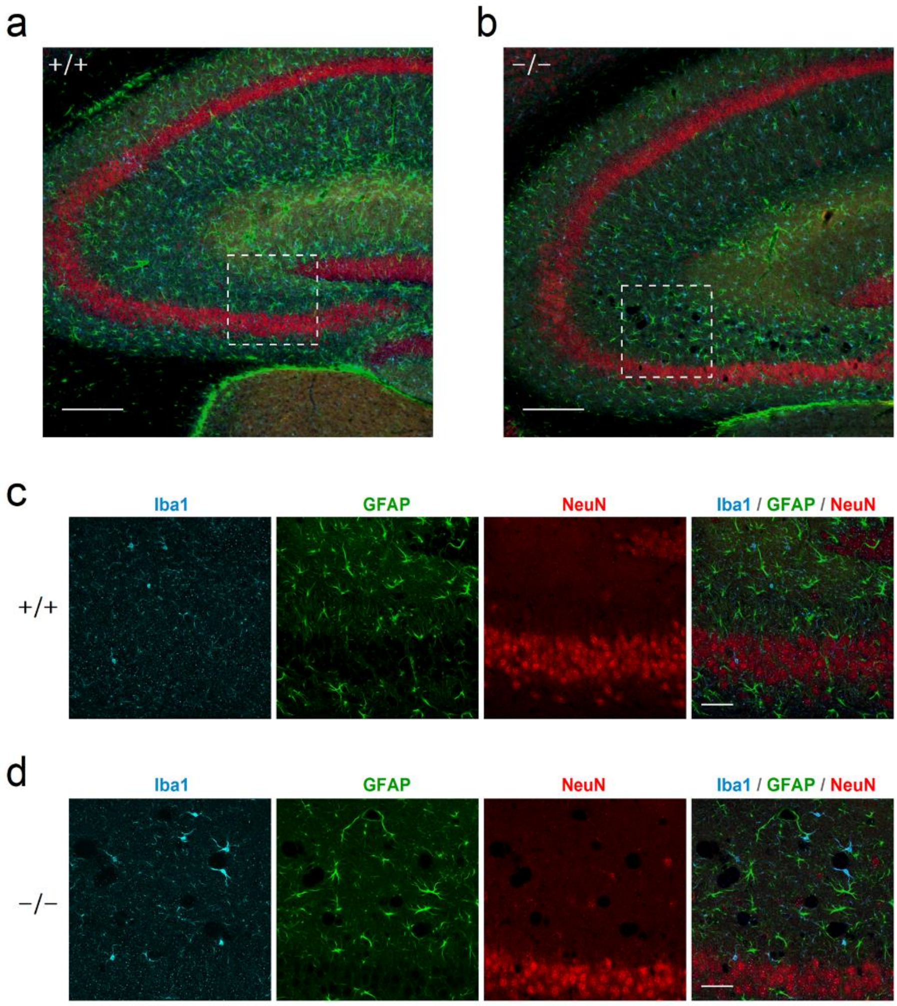

Supplementary Figure 7. Distribution of astrocytes and microglia in the hippocampal CA3 subfield of Xpnpep1$^{+/+}$ and Xpnpep1$^{-/-}$ mice. (a, b) Low-magnification images of Xpnpep1$^{+/+}$ (a) and Xpnpep1$^{-/-}$ (b) hippocampi showing distribution of NeuN-positive neurons, GFAP-positive astrocytes, and Iba1-positive microglia. Note the numerous vacuoles in the CA3 area of Xpnpep1$^{-/-}$ mice (b). (c, d) Compared to WT mice (c), Xpnpep1$^{-/-}$ mice (d) exhibited a higher number of microglia but fewer astrocytes in the CA3 region. However, accumulation of microglia or extension of astrocytic processes to vacuoles was not observed in the Xpnpep1$^{-/-}$ CA3 area. Scale bars, 200 μm (a and b) and 50 μm (c and d).

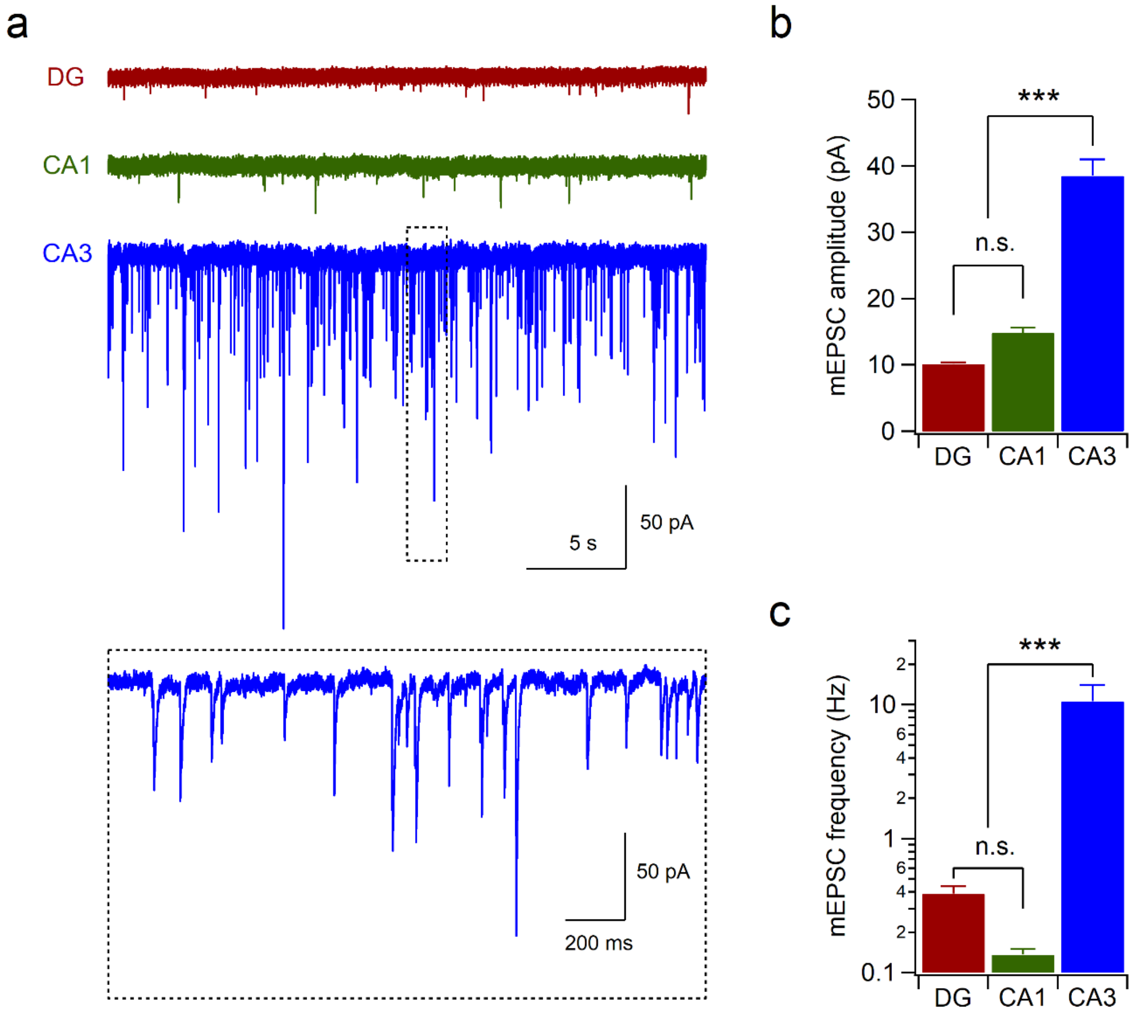

Supplementary Figure 8. Principal cells in the CA3 area receive more robust synaptic excitation than those in the DG and CA1 areas. (a) Representative mEPSC traces recorded from the DG granule cells (top), CA1 (middle), and CA3 (bottom) pyramidal cells of WT mice at the holding potential of −60 mV. The inset in the dotted box shows mEPSCs in the CA3 pyramidal cell on an expanded time scale. (b) The mean amplitudes of mEPSCs recorded from principal neurons in the DG, CA1, and CA3 areas are summarized. $F(2, 31) = 101.96$; n.s., not significant; *** $p < 0.001$; one-way ANOVA with Tukey's post-hoc test. (c) The frequency of mEPSCs onto CA3 pyramidal neurons was significantly higher than onto DG granule cells or CA1 pyramidal neurons. $F(2, 31) = 9.30$; n.s., not significant; *** $p < 0.001$; one-way ANOVA with Tukey's post-hoc test. n = 11 (DG), 12 (CA1), and 11 (CA3) cells from 3 WT mice (b, c).

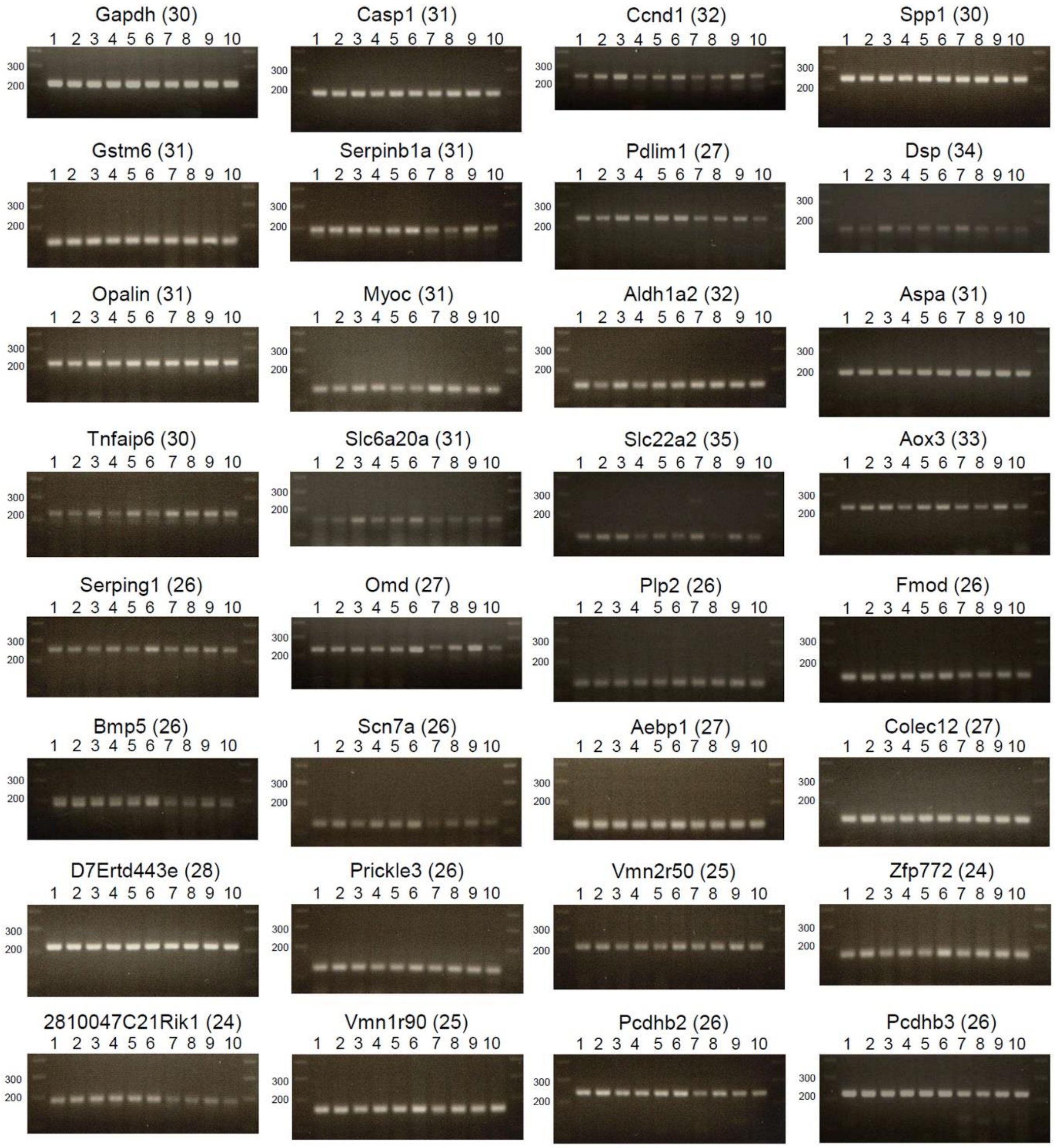

Supplementary Figure 9. Gel images of RT-PCR products amplified from Xpnpep1$^{+/+}$ and Xpnpep1$^{-/-}$ hippocampal mRNAs with gene-specific primers. The same hippocampal mRNAs used for qRT-PCR analyses shown in Figure 2 were also analyzed by conventional RT-PCR for checking primer specificity. Lane 1, +/+1; lane 2, −/−1; lane 3, +/+2; lane 4, −/−3; lane 5, +/+3; lane 6, −/−2; lane 7, +/+4; lane 8, −/−4; lane 9, +/+5; and lane 10, −/−5. The numbers in the parenthesis represent the number of PCR amplification cycles.

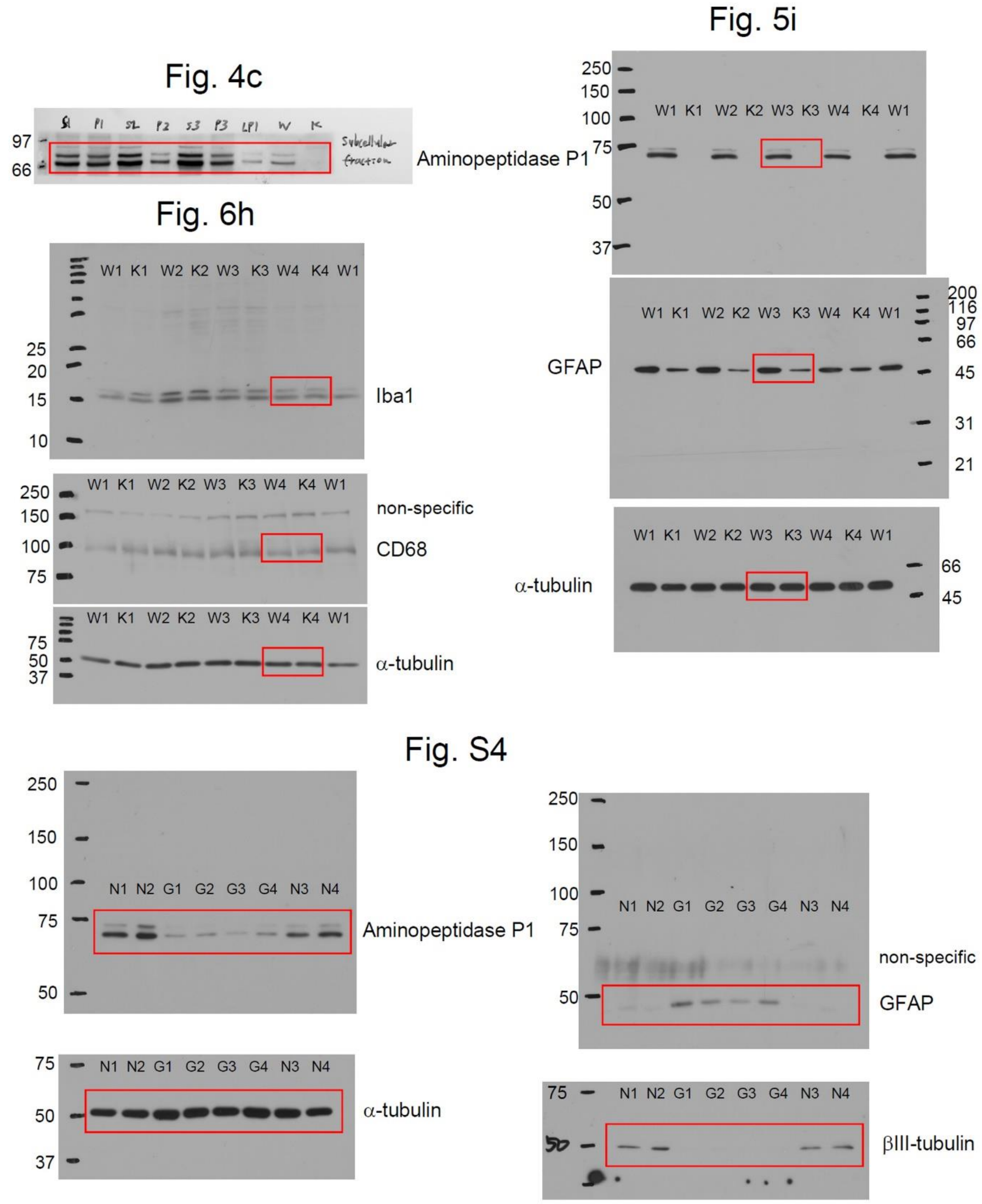

Supplementary Figure 10. Uncropped western blot images shown in the main and supplementary figures.

Supplementary Table 1. Primer sets used for quantitative PCR and RT-PCR

Gene symbol	Forward primer	Reverse primer	Amplicon (bp)	Tm (°C)
Vmn2r50	gctttggcattggtcttttc	ccaattgggtcctctaagca	216	60
Pcdhb3	gacctggatgtgggaagaaa	aagaccaggatgtggacctg	224	64
2810047C21Rik1	ccccagattgctagaaccaa	tcttcttggccaccagagtc	187	56
Zfp772	cctcctcgggtcttctaagg	gtgtgtatcatccggtgtcg	169	60
Vmn1r90	tggctgtgtccagcatctta	gtgctcaagacacaggtgga	160	60
Pcdhb2	ggtagaggaactcgctgcac	ccagaaaaacgggagaatga	239	66
Casp1	ccaggcaagccaaatcttta	cttgagggtcccagtcagtc	202	64
Ccnd1	agtgcgtgcagaaggagatt	cacaacttctcggcagtcaa	238	63
Myoc	tgcttcccaaatcttgaagg	ctccatacttgccagcgatt	137	60
Opalin	gatccagcgaagaagaacca	tcaggaggacggtaggtgtc	216	63
Aldh1a2	cggcatagacaagattgcat	ccaagtcagcatctgcaaaa	143	60
Aspa	ggtggagaagtgcaccagat	gcaacccatgttagaagtgg	202	60
Spp1	tgcacccagatcctatagcc	atccgaatggtgagattcgt	248	64
Gstm6	cctttgcagacttcctcgtc	cggctggtcttcatataggc	145	64
Serpinb1a	tgaagtgagcaaacgtggag	ttgacctttgacccactggt	193	64
Pdlim1	gcagacaacatgacgctcac	tcggatgagtagaggccagt	236	66
Dsp	tgctgcaaaattgctcagac	cctcatctctcggatcaagc	167	63
Tnfaip6	atttgaaggtggtcgtctcg	tcctttgcatgtgggttgta	225	60
Slc6a20a	gtgttgccagtgttgtggtc	tcctcatcgtagcctgtgtg	151	63
Slc22a2	acagggactggtcagcaaag	ggatcaggagcccaacagta	128	66
Serping1	tagccaacgagtccttcagc	ctgagaaggcgtggtagagc	249	60
Omd	gaccaagagccaaatgagga	tgcagtcacagcctcaatgt	231	63
Plp2	gttagcctgcaccagcttct	tcaccgacagggaggagtag	128	60
Fmod	cttccccacagccatgtact	atgtagagcgacccagagga	157	64
Bmp5	attttgggtttgcctcacag	attgggagaggctgggtatc	192	64
Scn7a	tctggaaatgctctccctgt	ttctagggcgaggaagcata	125	64
Aebp1	tgctcctgctcatgcaatac	cctcatagccatcagggttc	120	60

Colec12	acgagcagagcctgaaagac	gtgatgggctgtgtagctga	149	60
D7Ertd443e	ccacacgaatgacaatgagg	aactacagcggaggctgaga	218	60
Prickle3	cacatgggtcacttctgctg	catcacagtattccgcatgg	124	64
Aox3	agaaggcataggaagcacca	ggcagaaacttttcgcactc	230	64
Gapdh	acagcaactcccactcttccac	agttgggatagggcctctcttg	208	60
